# A dual-target molecular mechanism of pyrethrum repellency against mosquitoes

**DOI:** 10.1038/s41467-021-22847-0

**Published:** 2021-05-05

**Authors:** Feng Liu, Qiang Wang, Peng Xu, Felipe Andreazza, Wilson R. Valbon, Elizabeth Bandason, Mengli Chen, Ru Yan, Bo Feng, Leticia B. Smith, Jeffrey G. Scott, Genki Takamatsu, Makoto Ihara, Kazuhiko Matsuda, James Klimavicz, Joel Coats, Eugenio E. Oliveira, Yuzhe Du, Ke Dong

**Affiliations:** 1 Department of Entomology, Michigan State University, East Lansing, MI USA; 2 Department of Preventive Medicine and Public Health Laboratory Science, School of Medicine, Jiangsu University, Zhenjiang, Jiangsu China; 3 Department of Entomology, Universidade Federal de Viçosa, Viçosa, MG Brazil; 4 Department of Biology, Duke University, Durham, NC USA; 5 Institute of Pesticide and Environmental Toxicology, Zhejiang University, Hangzhou, China; 6 Institute of Health and Environment, Wenzhou Medical University, Wenzhou, China; 7 Department of Entomology, Cornell University, Ithaca, NY USA; 8 Department of Applied Biological Chemistry, Faculty of Agriculture, Kindai University, Nakamachi, Nara Japan; 9 Department of Entomology, Iowa State University, Ames, IA USA

**Keywords:** Neuroscience, Physiology

## Abstract

Pyrethrum extracts from flower heads of *Chrysanthemum* spp. have been used worldwide in insecticides and repellents. While the molecular mechanisms of its insecticidal action are known, the molecular basis of pyrethrum repellency remains a mystery. In this study, we find that the principal components of pyrethrum, pyrethrins, and a minor component, (E)-β-farnesene (EBF), each activate a specific type of olfactory receptor neurons in *Aedes aegypti* mosquitoes. We identify *Ae. aegypti* odorant receptor 31 (AaOr31) as a cognate Or for EBF and find that Or31-mediated repellency is significantly synergized by pyrethrin-induced activation of voltage-gated sodium channels. Thus, pyrethrum exerts spatial repellency through a novel, dual-target mechanism. Elucidation of this two-target mechanism may have potential implications in the design and development of a new generation of synthetic repellents against major mosquito vectors of infectious diseases.

## Abstract (web summary)

Although pyrethrum is used to repel insects for centuries, the underlying mechanism has been enigmatic. Liu et al. found that specific components of pyrethrum mediate repellency by targeting olfactory receptors and/or voltage-gated sodium channels in *Aedes aegypti* mosquitoes.

## Introduction

Mosquito-transmitted human diseases, such as malaria and dengue fever, represent significant burdens to global human health and cause considerable human suffering. One of the most effective measures to reduce disease transmission involves the use of insect repellents to prevent human contacts with mosquitoes. Pyrethrum extract from the dried and crushed flower heads of *Chrysanthemum (Tanacetum cinerariifolium*) began to be used as an insect repellent against biting arthropods thousands of years ago^1,2^. Today, *T, cinerariifolium* plants are grown commercially in many parts of the world, particularly in East Africa and Australia, for extraction of pyrethrum^3,4^ and pyrethrum and its synthetic analogs, pyrethroids, are key active ingredients in a variety of commercial insect-repelling products, such as mosquito coils, emanators, vaporizer mats, textile finishes, hessian strips/ribbons^5–13^. Pyrethrins, the major insecticidal components, are used to control a wide range of pests in both agricultural and non-agricultural settings and may pose harmful effects on bees when used outdoor. In addition, pyrethrum-producing *Chrysanthemum* spp. are recommended as “companion” plants to repel pest insects^14^.

Pyrethrins and pyrethroids exert potent insecticidal activities by hyper-activating insect voltage-gated sodium channels, thereby causing rapid paralysis, known as knockdown, and eventual lethality^15–17^. While the molecular mechanism of insecticidal lethal action of these compounds is well-established, the molecular mechanism(s) underlying the repellency elicited by pyrethrum and pyrethroids, at sublethal levels, remains a mystery. Various possibilities have been proposed, including contact-based repellency, spatial repellency, neuronal irritation, olfactory responses^11,18–26^. However, it is not clear how these possibilities relate to each other, and, most importantly, if olfactory responses are involved, what is the identity of the odorant receptor(s) responsible for pyrethrum repellency.

In this study, we took a combination of molecular genetics, electrophysiological and behavioral approaches to gain insights into pyrethrum repellency in *Aedes aegypti*, the primary vector of viruses that cause dengue fever, Zika, yellow fever and chikungunya. We aimed to address the following key questions: (i) Is pyrethrum repellency odorant receptor (Or)-mediated? (ii) If yes, which Or(s) is activated by pyrethrum? (iii) What component(s) of pyrethrum extract activates the Or(s)? and (iv) Is there a synergistic interaction among pyrethrum components in repellency? Our study resolved all these questions and revealed a novel, dual-target mechanism for insect repellency, involving dual activation of olfactory repellency pathways and voltage-gated sodium channels. The discovery has significant basic and practical implications in understanding the mechanisms and development of insect repellents against mosquitoes and other human disease vectors.

## Results

### Pyrethrum elicits spatial repellency

**Fig. 1:**
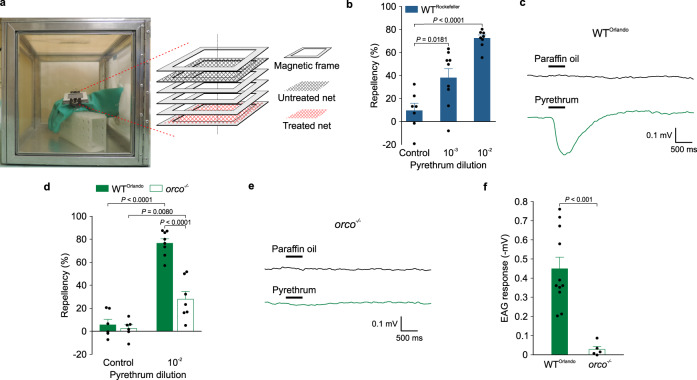
Pyrethrum elicits spatial repellency in *Ae. aegypti* mosquitoes. **a** A schematic depiction of the setup for the hand-in-cage assay as previously described^27^. **b** Dose-dependent pyrethrum repellency in Rockefeller (wild-type) mosquitoes (*n* = 7 cages for control; *n* = 9 cages for 10^−3^ (v v^−1^); *n* = 8 cages for 10^−2^ (v v^−1^) from 3 batches of mosquitoes; *t* = 2.68, *df* = 14, *P* = 0.0181 for control *vs*. pyrethrum at 10^−3^ and *t* = 9.58, *df* = 13, *P* < 0.0001 for control *vs*. pyrethrum at 10^−2^). **c** Representative EAG response traces of Orlando (wild-type) mosquitoes to pyrethrum (10^−2^ v v^−1^). **d** Repellency of pyrethrum (10^−2^ v v^−1^) against Orlando or *orco*^*−/−*^ mutant mosquitoes (*n* = 6 cages for each of the controls; *n* = 8 cages for pyrethrum in Orlando, *n* = 7 cages for pyrethrum in *orco*^*−/−*^, from 3 batches of mosquitoes; *t* = 11.74, *df* = 12, *P* < 0.0001 for control vs. pyrethrum in Orlando, *U* = 3, *P* = 0.008 for control vs. pyrethrum in *orco*^*−/−*^, and *t* = 6.53, *df* ^*=*^ 13, *P* < 0.0001, for Orlando *vs. orco*^*−/−*^ for pyrethrum). **e** Representative EAG response traces of *orco*^*−/−*^ mosquitoes to pyrethrum (10^−2^ v v^−1^). **f** Comparison of EAG responses in Orlando and *orco*^*−/−*^; *t* = 4.61, df = 14, *P* = 0.0004, *n* = 5 antennae for *orco*^*−/−*^, and *n* = 11 antennae for Orlando. Two-tailed unpaired student’s *t*-test or two-tailed Mann-Whitney Rank Sum test was used to compare each of two sets of data. Data are plotted as mean ± s.e.m. and dots denote the value of each repeat.

First, we determined whether pyrethrum elicits spatial repellency using a hand-in-cage assay^27^. In this behavioral assay, mosquitoes are attracted to a human hand in a modified glove (Fig. 1a) that has a screened window on the back of the glove. Mosquitoes would land on a piece of mesh secured on the top of the window. Between the top mesh and the hand is a second mesh, which was above the hand and treated with a test compound or solvent, but mosquitoes cannot make direct contact with the second mesh (Fig. 1a). The landing frequency of female Rockefeller mosquitoes (wild-type *Ae. aegypti*) onto the top mesh was significantly reduced when the second mesh was treated with pyrethrum (Sigma-Aldrich), and pyrethrum repellency increased in a dose-dependent manner (Fig. 1b). Similar spatial pyrethrum repellency was also observed in female Kisumu mosquitoes (wild-type *Anopheles gambiae*) (Supplementary Fig. 1a). These results clearly show contact-independent, spatial repellency of pyrethrum against mosquitoes.

Next, we examined whether spatial repellency by pyrethrum depends on the mosquito olfaction system. We found that pyrethrum elicits electroantennagram (EAG) responses in *Ae. aegypti* and *An. gambiae* mosquitoes (Fig. 1c; Supplementary Fig. 1b). Pyrethrum-induced EAG response in *Ae. aegypti* was also reported recently in another study^24^. In contrast, no response to pyrethrum was detected from mosquito maxillary palps, another major olfactory organ in insects (Supplementary Fig. 1c). These results indicate that the olfactory receptor neurons (ORNs) in the antennae of *Ae. aegypti* and *An. gambiae* mosquitoes are likely responsible for sensing pyrethrum.

In insects, in addition to individual Ors that are responsible for recognizing specific odorants, an obligate co-receptor (Orco) is required for detection of diverse odorants^28–30^ and can be used to infer whether insect attraction or avoidance response is Or-mediated. No obvious degenerate DAPI-stained ORNs were detected from *orco*^*−/−*^ *Anopheles* mosquitoes^31^. We found that pyrethrum repellency was significantly reduced in *orco*^*−/−*^ *Ae. aegypti* mosquitoes, compared to the wild-type *Ae. aegypti* strain Orlando, from which the *orco*^*−/−*^ mutant was generated^32^ (Fig. 1d). The EAG signals from *orco*^*−/−*^ mutant mosquitoes were also dramatically reduced compared to that from Orlando mosquitoes (Fig. 1e, f). These results indicated that spatial repellency by pyrethrum is largely Or-mediated.

### Pyrethrum activates specific olfactory receptor neurons and odorant receptors

**Fig. 2:**
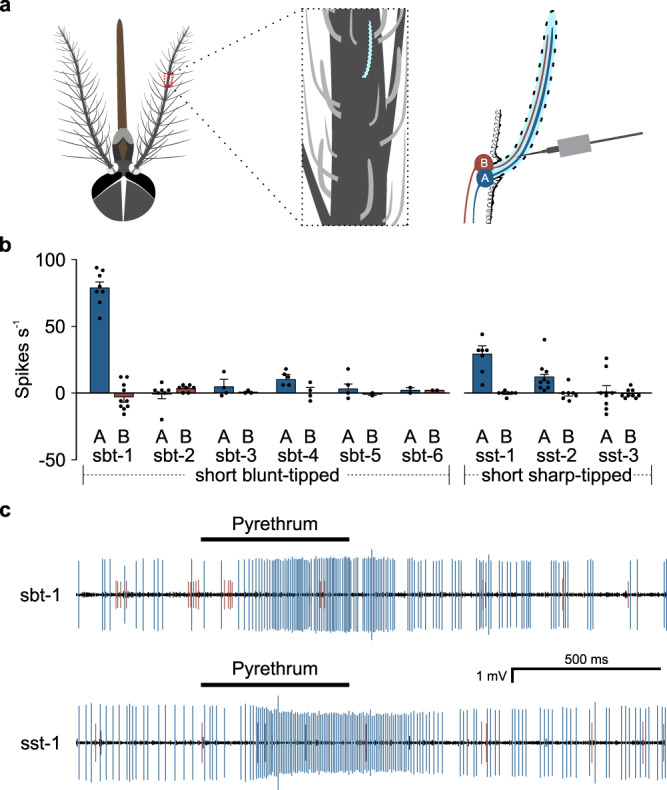
Identification of pyrethrum-responsive sensilla in *Ae. aegypti* antennae. **a** A schematic drawing illustrating single sensillum recording (SSR) from *Ae. aegypti* mosquito antennae. **b** SSR responses of short sharp-tipped (sst) and short blunt-tipped (sbt) sensilla to pyrethrum (10^−2^ v v^−1^) (*n* = 8 sensilla for sbt-1; *n* = 7 for sbt-2, sst-1 and sst-2; *n* = 3 for sbt-3 and sbt-4; *n* = 2 for sbt-5 and sbt-6; *n* = 9 for sst-3). **c** Representative SSR traces indicating increased firing of A neurons of sbt-1 sensilla (*n* = 8 sensilla) or sst-1 sensilla (*n* = 7 sensilla) in response to pyrethrum (10^−2^ v v^−1^) in Rockefeller mosquitoes. Data are plotted as mean ± s.e.m. and dots denote the value of each repeat.

Insect Ors are mainly expressed in olfactory receptor neurons (ORNs) in antenna^29,33–35^. Three major morphologically distinct types of antennal trichodae sensilla are recognized in *Ae. aegypti* antennae: short sharp-tipped (sst), long sharp-tipped (lst), and short blunt-tipped (sbt)^36,37^. Each *Ae. aegypti* sensillum houses two neurons: The neuron that generates larger spikes (i.e., action potentials) is called the A neuron and the neuron that produces smaller spikes is called the B neuron. To identify which mosquito ORN(s) responds to pyrethrum, we performed single sensillum recordings (SSR) of antennal olfactory sensilla of Rockefeller mosquitoes in response to a panel of odorants including pyrethrum, (±)-citronellal, geranyl acetate and (-)-borneol (Fig. 2a; Supplementary Fig. 2), most of which are plant-derived mosquito repellents. We identified three responsive sst sensilla, sst-1 to sst-3, and six responsive sbt sensilla, sbt-1 to sbt-6, based on their response profiles to the odorants in our panel (Supplementary Fig. 2). lst sensilla did not respond to any odorants in our panel.

We noted that sbt-1 and sst-1 sensilla were most responsive to pyrethrum (Fig. 2b). In particular, the A neuron in the sbt-1 sensilla was excited by pyrethrum, while the B neuron was not (Fig. 2c). Interestingly, the sbt-1A neuron also exhibited strong excitatory responses to (±)-citronellal and geranyl acetate (Supplementary Fig. 2), which are key components of other natural insect repellents. In contrast, the sbt-1B neurons were highly sensitive to indole and toluene (Supplementary Fig. 2). For sst-1 sensilla, pyrethrum specifically activated sst-1A neurons, but not sst-1B (Fig. 2c). In addition, sst-1A was also activated by (±)-citronellal, camphor and eucalyptol (Supplementary Fig. 2), which are known repellents against mosquitoes. Furthermore, similar response profiles of sbt-1 and sst-1 to pyrethrum and other volatiles were also detected from Orlando mosquitoes (Supplementary Fig. 3). In addition, pyrethrum also weakly activated sbt-4A and sst-2A (Fig. 2b), suggesting that the olfactory response to pyrethrum is likely not exclusively dependent on sbt-1A and sst-1A. Furthermore, additional sensilla type(s) besides the ones we identified in Supplementary Fig. 2 could respond to pyrethrum.

**Fig. 3:**
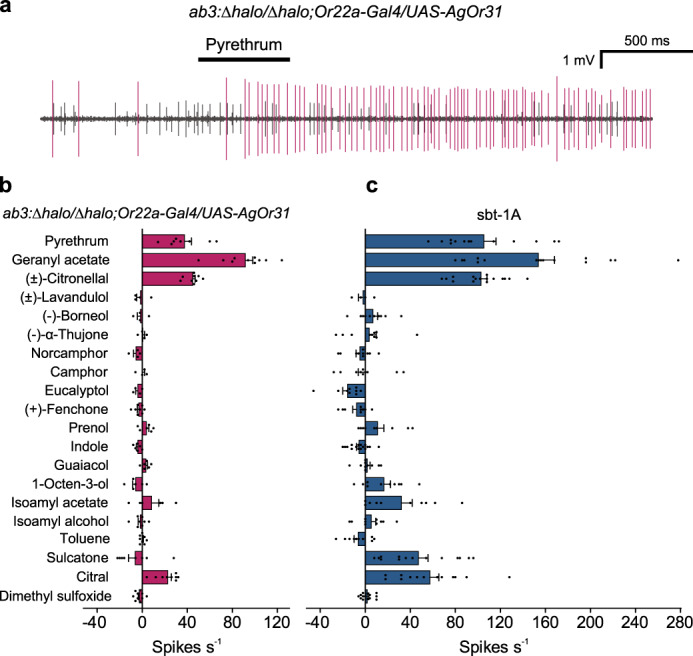
Identification of pyrethrum-responsive AgOr31 from *An. gambiae*. **a** A representative single sensillum recording (SSR) trace (*n* = 8 sensilla) from *Drosophila* ab3A empty neurons expressing AgOr31 to pyrethrum (10^−2^ v v^−1^). *An. gambiae* AgOrs were expressed heterologously in the empty neuron (i.e., the ab3A neuron of the ab3 sensilla) in *D. melanogaster*. **b** Odorant response profiles from AgOr31 (*n* = 4 sensilla). **c** Odorant response profiles from sbt-1A neuron in *Ae. aegypti* (*n* = 16 sensilla/mosquitoes). Data are plotted as mean ± s.e.m. and dots denote the value of each repeat.

Having identified sbt-1 and sst-1 as primary ORNs that are responsive to pyrethrum, we next attempted the challenging goal of identifying specific Or(s) that are involved. For this purpose, we took advantage of the availability of an indexed library of *An. gambiae* Ors (AgOrs)^38^, and expressed each of the 50 AgOrs in the “ab3 empty neuron” system of *Drosophila melanogaster*, in which the endogenous Or gene *Or22a* in the ab3 sensillum was deleted^39^. We examined SSR response from the resulting chimera ab3 sensillum expressing individual AgOrs in the *D. melanogaster* antenna to the same panel of odorants used in the analysis of *Ae. aegypti* sensilla. We found that pyrethrum activated AgOr31 (Fig. 3a), AgOr20, AgOr53 and AgOr76 (Supplementary Fig. 4a). As shown in Fig. 3b, c (also Supplementary Fig. 4b), the odorant response profile of the sbt-1A neuron matches remarkably well with that of AgOr31. The odorant response profiles of the other three pyrethrum-activated AgOrs, AgOr20, AgOr53 and AgOr76 did not match with the response profiles of the sbt-1A or sst-1A neurons (Supplementary Fig. 4b, c). Phylogenetic analysis of Or protein sequences from seven mosquito species, including *Ae. aegypti*, *Ae. albopictus*, *An. gambiae*, *An. coluzzii*, *An. dirus*, *An. albimanus* and *Culex quinquefaciatus*, revealed that AaOr31 from *Ae. aegypti*, AgOr31 from *An. gambiae* and related Ors from the other five mosquito species fall into a single distinct clade (Supplementary Fig. 5). Therefore, AgOr31 and AaOr31 are most likely orthologous based on matching odorant response profiles (Fig. 3b, c) and the close phylogenetic relationship. In contrast, no orthologues of *AgOr20*, *AgOr53* or *AgOr76* are found in *Ae. aegypti*, *Ae. albopictus* or *Cx. quinquefaciatus*, suggesting that *Or31* represents a widely conserved mosquito *Or* gene, whereas *AgOr20*, *AgOr53* and *AgOr76* likely belong to a family of *Anopheles*-specific *Or* genes (Supplementary Fig. 5). Future research using *Drosophila* empty neurons expressing AaOr31 and Or31 orthologs from other five mosquito species could further examine the proposed orthologous relationship across the major mosquito species.

### (*E*)-β-farnesene (EBF) activates pyrethrum-responsive Or31

**Fig. 4:**
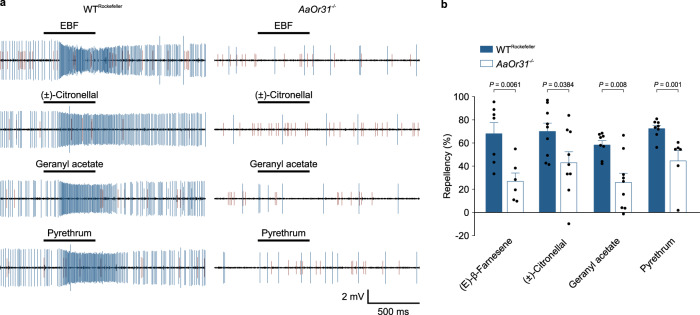
AaOr31-mediated (E)-β-farnesene (EBF)/pyrethrum repellency in *Ae. aegypti*. **a** Representative SSR traces from short blunt-tipped (sbt)-1 sensilla in Rockefeller (wild-type) and *AaOr31*^*−/−*^ mosquitoes in response to EBF (*n* = 10 sensilla for Rockefeller and *n* = 7 sensilla for *AaOr31*^*−/−*^), (±)-citronellal (*n* = 10 sensilla for Rockefeller and *n* = 8 sensilla for *AaOr31*^*−/−*^), geranyl acetate (*n* = 10 sensilla for Rockefeller and *n* = 8 sensilla for *AaOr31*^*−/−*^) and pyrethrum (*n* = 10 sensilla for Rockefeller and *n* = 8 sensilla for *AaOr31*^*−/−*^) at 10^−2^ dilution. **b** Repellency in *AaOr31*^*−/−*^ compared with that of Rockefeller mosquitoes to EBF (*t* = 3.38, *df* = 11, *P* = 0.0061; *n* = 6 cages for *AaOr31*^*−/−*^ and *n* = 7 cages for Rockefeller from 3 batches of mosquitoes), (±)-citronellal (*t* = 2.26, *df* = 16, *P* = 0.0384, *n* = 9 cages for *AaOr31*^*−/−*^ and *n* = 9 cages for Rockefeller from 4 batches of mosquitoes), geranyl acetate (*U* = 8, *P* = 0.008, *n* = 8 cages for Rockefeller, *n* = 9 cages for *AaOr31*^*−/−*^ from 4 batches of mosquitoes) and pyrethrum (*U* = 1, *P* = 0.001, *n* = 6 cages for *AaOr31*^*−/−*^, *n* = 8 cages for Rockefeller from 3 batches of mosquitoes) at 10^−2^ dilution. Two-tailed unpaired student’s *t*-test or two-tailed Mann–Whitney Rank Sum test was used to compare each of two sets of data. Data are plotted as mean ± s.e.m. and dots denote the value of each repeat.

Given that pyrethrum is an extract containing six insecticidal esters (i.e., collectively known as pyrethrins) as major components and several phytochemicals as minor components, including (*E*)-β-farnesene (EBF), β-cubebene, ethyl palmitate and ethyl linoleate^40–42^, we next addressed the important question of which component(s) in pyrethrum activates Or31 by conducting SSR of AgOr31-expressing chimera ab3 sensilla to these individual components. Remarkably, EBF (Sigma-Aldrich) activated not only AgOr31 expressed in *Drosophila* ab3 empty neurons (Supplementary Fig. 6a), but also sbt-1A neurons in *Ae. aegypti* (Fig. 4a). None of the insecticidal esters, ethyl palmitate or ethyl linoleate activated AgOr31 (Supplementary Fig. 6b). β-cubebene was not commercially available to be examined in our study. Furthermore, EBF elicited spatial repellency at the 10^−2^ dilution (i.e., 114 μg cm^−2^) (Fig. 4b). It should be pointed out that a previous study^43^ did not observe repellency by EBF using the concentration of ~10 μg cm^−2^. Consistent with their result, we did not detect repellency by EBF at the 10^−3^ dilution (i.e., 11.4 μg cm^−2^) (Supplementary Fig. 6c), indicating that EBF repellency is concentration-dependent.

### Knockout of *AaOr31* reduced pyrethrum and (*E*)-β-farnesene repellency

Next, we asked whether *Ae. aegypti* Or31 (AaOr31) contributes to mosquito avoidance behavior to pyrethrum, EBF and other volatiles that activate AaOr31. For this purpose, we generated *AaOr31* knockout (*AaOr31*^*−/−*^*)* mosquitoes in *Ae. aegypti* strain Rockefeller using the CRISPR-Cas9 technology. Genotyping revealed that the *AaOr31* gene of *AaOr31*^*−/−*^ mosquitoes carried two deletions in exon 2 of *AaOr31*, which resulted in a premature stop codon (Supplementary Fig. 7a). In *AaOr31*^*−/−*^ mosquitoes, we were able to identify sbt-1 sensilla by the response of sbt-1B neurons to both indole and toluene since none of other types of sbt sensilla (sbt2-6) responds to both indole and toluene (Supplementary Fig. 2). As expected, the response of sbt-1B neurons in *AaOr31*^*−/−*^ mosquitoes to both indole and toluene remained intact (Supplementary Fig. 7b). Strikingly, the response of sbt-1A neurons to EBF, geranyl acetate or (±)-citronellal was completely abolished in *AaOr31*^*−/−*^ mosquitoes (Fig. 4a). Furthermore, the repellency by EBF, (±)-citronellal and geranyl acetate were all significantly reduced in *AaOr31*^*−/−*^ mosquitoes compared to wild-type mosquitoes (Fig. 4b). Similarly, knockout of *AaOr31* abolished the response of sbt-1A neurons to pyrethrum (Fig. 4a) and reduced pyrethrum repellency (Fig. 4b). Knockout of *AaOr31* did not alter the response profile of sst-1A to pyrethrum and other odorants examined (Supplementary Fig. 7c). Collectively, these data showed that AaOr31-mediated olfactory pathway is an important component of the pyrethrum repellency mechanism.

### Pyrethrins elicit repellency by activating specific olfactory receptor neurons and sodium channels

**Fig. 5:**
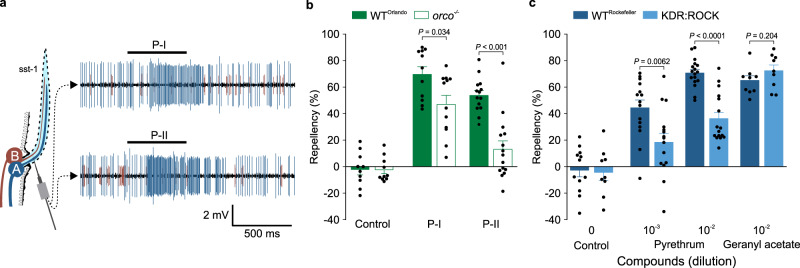
Pyrethrin repellency is the result of activation of both Or(s) and sodium channels. **a** Representative single sensillum recording (SSR) traces of short sharp-tipped (sst)-1 sensilla responding to pyrethrin I (P-I; *n* = 8 sensilla) and pyrethrin II (P-II; *n* = 6 sensilla) at the 10^−1^ dilution from Rockefeller mosquitoes. **b** Repellency elicited by P-I and P-II at the 10^−3^ dilution in Orlando (wild-type) and *orco*^*−/−*^ mosquitoes (*n* = 10 cages for Orlando control and *orco*^*−/−*^ control from 2 batches of mosquitoes; P-I: *U* = 31, *P* = 0.034, *n* = 11 cages for Orlando and *n* = 12 cages for *orco*^*−/−*^ from 3 batches of mosquitoes; P-II: *U* = 15, *P* < 0.001, *n* = 14 cages for Orlando and *n* = 15 cages for *orco*^*−/−*^ from 4 batches of mosquitoes). **c** Repellency by pyrethrum and geranyl acetate in KDR:ROCK mosquitoes compared to that in Rockefeller mosquitoes (*n* = 10 cages for control in Rockefeller and *n* = 9 cages for control in KDR:ROCK from 2 batches of mosquitoes; pyrethrum at the 10^−3^ dilution: *t* = 2.96, *df* = 28, *P* = 0.0062, *n* = 15 cages for Rockefeller and *n* = 15 cages for KDR:ROCK from 4 batches of mosquitoes; pyrethrum at the 10^−2^ dilution: *t* = 6.56, *df* = 31, *P* < 0.0001, *n* = 17 cages for Rockefeller and *n* = 16 cages for KDR:ROCK from 4 batches of mosquitoes; geranyl acetate: *t* = 1.32, *df* = 16, *P* = 0.204, *n* = 9 cages for each strain from 2 batches of mosquitoes). Two-tailed unpaired student’s *t*-test or two-tailed Mann-Whitney Rank Sum test was used to compare each of two sets of data. Data are plotted as mean ± s.e.m. and dots denote the value of each repeat.

We noted, however, that more than 40% of pyrethrum repellency remained in *AaOr31*^*−/−*^ mosquitoes, as shown in Fig. 4b. Furthermore, the percentage of EBF in commercial pyrethrum extracts is generally very low (ranging from 1.25% to 1.97% based on our analysis of the pyrethrum extracts used in this study), which alone would not be sufficient to evoke repellency. These results suggest additional mechanism(s) underlying pyrethrum repellency. As shown in Fig. 2b, c, besides sbt-1, sst-1 was the only other type of sensillum that strongly responded to pyrethrum. We predicted that additional component(s) in pyrethrum likely elicit repellency by activating the sst1-associated olfactory pathway. Indeed, we found that pyrethrin I (P-I) and pyrethrin II (P-II), two major components purified from pyrethrum extracts (Supplementary Fig. 8), activate sst-1A neurons in Rockefeller mosquitoes (Fig. 5a). A similar response of sst-1A neurons to pyrethrin I was also observed in *AaOr31*^*−/−*^ mosquitoes (Supplementary Fig. 7c). Both P-I and P-II elicited spatial repellency (Fig. 5b) and pyrethrin repellency was significantly reduced in *orco*^*−/−*^ mosquitoes (Fig. 5b), suggesting an important role of sst-1A neurons in pyrethrin-induced repellency. In addition, because pyrethrins are known to hyper-activate voltage-gated sodium channels that are critical for electrical signaling^44,45^, we examined a possible involvement of activation of sodium channels in pyrethrin repellency. For this purpose, we used the KDR:ROCK mutant line, which is near isogenic to the wild-type Rockefeller strain and resistant to pyrethrum due to two defined mutations in the sodium channel gene^46^ (Supplementary Fig. 9a). Compared with wild-type Rockefeller mosquitoes, we found that pyrethrin/pyrethrum repellency was reduced in KDR:ROCK mosquitoes (Fig. 5c; Supplementary Fig. 9b). However, GA repellency was not reduced in KDR:ROCK mosquitoes (Fig. 5c). These results indicate the involvement of sodium channel activation in pyrethrin repellency. Thus, pyrethrins contribute to pyrethrum repellency via two mechanisms: (i) activation of sst-1A-associated olfactory pathway and (ii) hyper-activation of voltage-gated sodium channels.

### Synergism between AaOr31-mediated repellency and sodium channel-activated repellency

**Fig. 6:**
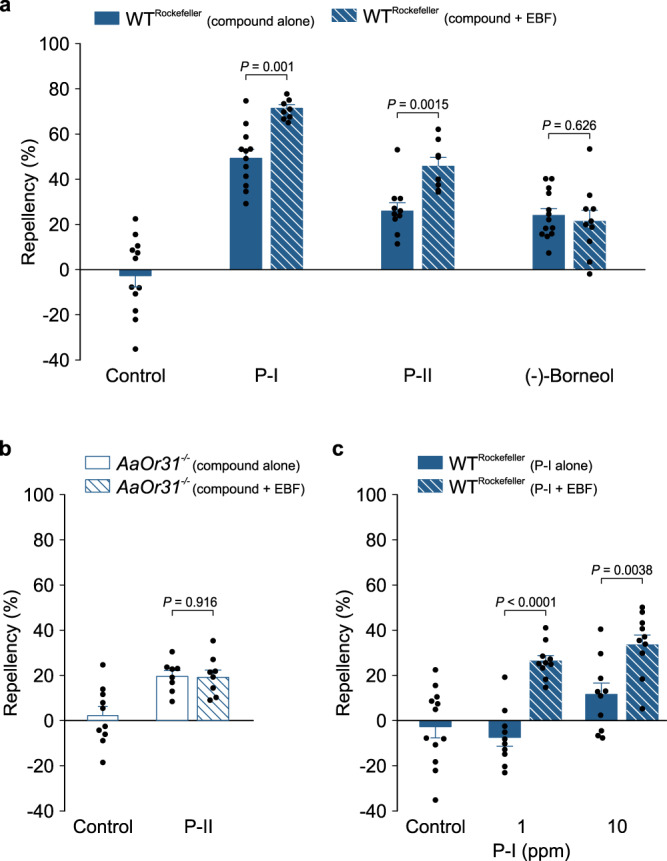
Pyrethrins-induced repellency is synergized by (E)-β-farnesene (EBF) via activation of both AaOr31 and sodium channels. **a** Effect of EBF (4 ppm) on pyrethrin I (P-I; 1000 ppm), pyrethrin II (P-II; 1000 ppm) and (-)-borneol (100 ppm) repellency in Rockefeller (*n* = 10 cages for control from 2 batches of mosquitoes; *U* = 6, *P* = 0.001*; n* = 8 cages for P-I + EBF and *n* = 12 cages for P-I alone from 3 batches of mosquitoes; *t* = 3.82, *df* = 16, *P* = 0.0015; *n* = 10 cages for P-II alone and *n* = 8 cages for P-II + EBF from 3 batches of mosquitoes; and *t* = 0.49, *df* = 21, *P* = 0.626, *n* = 13 cages for (-)-borneol alone and *n* = 10 cages for (-)-borneol + EBF from 3 batches of mosquitoes. **b** No effect of EBF (4 ppm) on P-II (1000 ppm) repellency in *AaOr31*^*−/−*^ mosquitoes (*n* = 10 cages for control from 2 batches of mosquitoes; *t* = 0.11, *df* = 14, *P* = 0.916, *n* = 8 cages for P-II alone and *n* = 8 for P-II + EBF from 2 batches of mosquitoes). **c**, Effect of EBF (10 ppm) on repellency by P-I (1 ppm and 10 ppm) in Rockefeller (*n* = 10 cages for control; P-I at the 1 ppm: *t* = 7.34, *df* = 18, *P* < 0.0001, *n* = 10 for P-I alone and *n* = 10 for P-I + EBF from 2 batches of mosquitoes; P-I at 10 ppm: *t* = 3.32, *df* = 18, *P* = 0.0038, *n* = 10 cages for P-I alone and *n* = 10 cages for P-I + EBF from 2 batches of mosquitoes). EBF alone at 4 ppm and 10 ppm did not elicit repellency in Rockefeller or *AaOr31*^*−/−*^ mosquitoes (Supplementary Fig. 6d). Two-tailed unpaired student’s *t*-test or *t*wo-tailed Mann-Whitney Rank Sum test was used to compare each of two sets of data. Data are plotted as mean ± s.e.m. and dots denote the value of each repeat.

Identification of sst-1A and sbt-1A OSNs and voltage-gated sodium channels as targets of pyrethrum repellency raised a fundamental question with respect to the relationship between activation of sodium channels and olfactory pathways in pyrethrum-mediated mosquito repulsion. To address this question, we examined mosquito repellency in response to pyrethrins I/II or EBF alone or in combination. Remarkably, EBF enhanced pyrethrin I or pyrethrin II repellency at a concentration of as low as 4 ppm (Fig. 6a), whereas EBF alone at these concentrations did not evoke any repellency (Supplementary Fig. 6c). Importantly, the enhancement was specific to pyrethrins, as no enhancement in repellency was observed between EBF and another plant-derived repellent, (-)-borneol (Fig. 6a). Although, like pyrethrins, (-)-borneol activates sst-1A neurons (Supplementary Fig. 2) and elicits Orco-dependent repellency (Supplementary Fig. 10a), it cannot activate sodium channels (Supplementary Fig. 10b), suggesting that EBF enhancement may be specific to sodium channel-activating chemicals, such as pyrethrins. Furthermore, the enhancement was abolished in *AaOr31*^*−/−*^ mosquitoes (Fig. 6b), indicating that pyrethrins enhance AaOr31-dependent repellency evoked by EBF. Importantly, the synergism between pyrethrins and EBF could be observed even when the concentration of pyrethrin I was reduced to 10 ppm and 1 ppm which alone did not elicit repellency (Fig. 6c). Collectively, these results strongly suggest that hyper-activation of sodium channels by pyrethrins, together with AaOr31-mediated repellency by EBF, produce a potent synergism that could explain the pyrethrum repellency against mosquitoes. The potentiation of AaOr31-mediated repellency by pyrethrins raises the possibility of a broad effect of sodium channel hyper-activation on the mosquito olfactory system. We therefore examined a possible effect of pyrethrins on the activities of odorants other than EBF. We found that camphor and eucalyptol, which each activate multiple types of *Ae. aegypti* sensilla in both Rockefeller and Orlando mosquitoes (Supplementary Fig. 2), also evoked spatial repellency in both strains (Supplementary Fig. 11c and 12). The magnitude of repellency by eucalyptol and camphor, as well as by P-I and P-II (Figs. 5 and 6), was greater in Orlando mosquitoes compared with that in Rockefeller mosquitoes, which likely reflect natural variations between the two strains. Repellency by camphor and eucalyptol was Orco-dependent as reduced repellency was observed in *orco*^*−*/*−*^ mosquitoes (Supplementary Fig. 10c). Furthermore, P-I enhanced repellency by camphor and eucalyptol (Supplementary Fig. 11), confirming a broad effect of pyrethrins on repellency by other mosquito repellents. Next, we investigated whether the pyrethrin/EBF synergism occur at the olfactory sensory level. Specifically, we evaluated the activity of sbt-1 neurons in response to EBF alone or co-application with P-I. As expected, EBF evoked increased firing of stb1A neurons, but co-application of P-I and EBF did not enhance the EBF-evoked activity (Supplementary Fig. 12a, b). In addition, P-I did not alter the baseline firing of sbt-1 neurons in Rockefeller or AaOr31^*−/−*^ mosquitoes (Supplementary Fig. 12c), suggesting that sodium channels in these ORNs are not sensitive to P-I. Collectively, these results suggest that pyrethrin potentiation of Or-mediated repellency does not occur at the olfactory sensory level, pointing to its effect at another step, possibly at the central neural processing level.

## Discussion

In this study, we provide strong evidence that pyrethrum exerts spatial repellency through a novel, dual-target mechanism. A minor component of pyrethrum, (*E*)-β-farnesene (EBF), activates AaOr31, while Or31-mediated repellency is significantly synergized by pyrethrins, which are activators of voltage-gated sodium channels and the principal components of pyrethrum. It is remarkable that, centuries ago, humans unknowingly exploited a hidden potent synergism between activation of sodium channels by pyrethrins and activation of AaOr31 by EBF in a natural plant extract against insect bites. Elucidation of this two-target mechanism for a popular natural insect repellent has significant implications in the design of a new generation of synthetic repellent mixtures against major mosquito vectors of infectious human diseases, including malaria, dengue and Zika.

AaOr31 is conserved in all three major disease-transmitting mosquito genera, *Aedes, Anopheles* and *Culex* (Supplementary Fig. 5). To our knowledge, Or31 in the first conserved mosquito odorant receptor that has been demonstrated to mediate repellency. Furthermore, for this first time we show that, besides acting on sodium channels, pyrethrins also directly activate Or(s) located in sst-1A neurons, although the identity of pyrethrin-activated Or(s) remains to be determined. We speculate that the EBF-pyrethrins synergism in pyrethrum repellency is likely the result of action of pyrethrins on hypersensitive sodium channels which either directly enhances the activity of EBF/AaOr31-mediated repellency and/or affect another neural circuit(s) that controls host finding. Exactly how hyper-activation of sodium channels enhances Or-mediated repellency and/or inhibit host finding awaits future investigations. Previous research revealed extensive alternative splicing and RNA editing of the sodium channel transcript, which produces a wide range of functional diversity of insect sodium channels in *Drosophila* and cockroach, including differential sensitivities to pyrethroids^47^. It is likely that pyrethrins at non-lethal concentrations of pyrethrins activate pyrethrin–hypersensitive sodium channel variants expressed in certain neural circuits, which leads to depolarization of membrane potential and influences the activity of neural circuits in response to EBF and other Or-activating repellents. Our SSR recordings did not detect any effect of pyrethrin I on EBF-elicited response of sbt-1A neurons, suggesting that sodium channels in the ORNs per se were not the targets of pyrethrins. Functional identification and localization of such pyrethrin–hypersensitive sodium channel variants will be necessary to advance further mechanistic understanding of the pyrethrin-EBF synergism in repellency.

The discovery of a two-target mechanism in pyrethrum repellency will likely stimulate future development of a new generation of durable and wide-spectrum insect repellents. Indeed, citronellyl cyclobutanecarboxylate (CCBC) was recently developed as a promising synthetic citronellol-derived repellent with improved stability and other physiochemical properties. As shown in Supplementary Fig. 13, we discovered that CCBC activates AgOr31 and stb-1A neurons and that CCBC repellency is significantly reduced in *AaOr31*^*−/−*^ mosquitoes. Therefore, we have accidentally identified AaOr31 as a major target of CCBC, in addition to pyrethrum. This illustrates the promising nature of Or31 for new repellent discoveries.

## Methods

### Mosquito strains

Five *Ae. aegypti* mosquito strains were used: Rockefeller and Orlando are two wild-type strains and *orco*^*−/−*^ (*orco*^16^) mosquito is a mutant mosquito strain with the *orco* gene mutated^32^ (BEI Resources, NIAID, NIH). KDR:ROCK is a pyrethroid-resistant strain^46^. The *AaOr31*^*−/−*^ mutant strain was generated in this study using the CRISPR-Cas9 technology and was backcrossed with parental Rockefeller mosquitoes for four generations to generate a near-isogenic line for functional analysis. One *An. gambiae* strain was used: Kisumu (BEI Resources, NIAID, NIH).

### Hand-in-cage assay

The hand-in-cage assay was similar to the hand-in-glove assay by Boyle et al.^27^. The setup for the hand-in-cage assay includes a 30 cm × 30 cm × 30 cm mosquito cage (BioQuip, Rancho Dominguea, CA), with a mounted digital camera and a human hand in a modified glove with a screened window. The digital camera (e-con Systems Inc, San Jose, CA, model: e-CAM51A) for video recording is mounted on the cage top and connected to a laptop computer. A nitrile rubber glove (Ansell Protective Products, Coshoton, OH, part number: 37-155) was cut on the back side of the glove to create a window (6 cm×5 cm) (Fig. 1a). A piece of magnetic frame (slightly larger than the dimension of the window) was glued onto the cut window which was used as a base for stacking more magnetic window frames (Fig. 1a; also further explained below). One piece of test compound-treated polyester netting (Shason Textile Inc., part number: WS-B532-111, white; 6.5 cm×5.5 cm) was placed on this fixed magnetic frame, which was ~3.0 mm above the glove. The second piece of the netting was untreated and placed ~8.0 mm above the treated net using a stack of four magnetic frames. The stacked magnetic frames were further secured with a binder clip. The stacking creates sufficient space between the treated net and the untreated net so that mosquitoes that land on the open window were not able to contact the treated net or contact and pierce the skin of the hand in the glove. The hand makes no contact with the treated net.

The assay was run in a room with relative humidity around 50% and temperature between 27 °C to 30 °C. Twenty-four hours before an assay, four to nine days-old females (about 40, mated, non-blood fed) were transferred into a mosquito cage. The cage was kept in an incubator where mosquitoes were provided only with water in a cotton ball placed on the top of the cage. Immediately before the assay, one researcher (i.e., tester) treated a piece of netting with 500 µl test compound dissolved in acetone in a glass Petri dish in an adjacent room. Acetone served a control. After letting acetone evaporate (~7 min), the researcher assembled and put on a modified glove. In the meantime, a lab assistant transferred the prepared cage from the incubator to a bench in the assay room. Both personnel avoided use of any hand lotions and cosmetic products and wore white lab coats and gloves. The hand in the modified glove was introduced into the cage to initiate the assay. Mosquitoes landing on the test window was recorded by the digital camera for five minutes. The number of mosquitoes landing during the second to fifth minutes was counted and recorded. For each cage, solvent (acetone) control was tested first and then followed with a treatment. The time interval of assays between control and treatment was at least 1.5 h, allowing the mosquitoes to fully recover and residual vapors from experiments to be ventilated out of the room. Controls, which represent mosquito baseline activity in response to solvent, were from two trials of solvent 1.5 h apart to make sure that mosquitoes continue landing in the second trial at the same rate. Data from any cage that gave a low landing number in a control trial were discarded. Percentage repellency was determined for each cage using the following equation: Percentage repellency = [1- (cumulative number of mosquitoes landed on the window of treatment /cumulative number of mosquitoes landed on the window of solvent treatment)] x 100). Each experiment was repeated at least by two different testers.

Once the assay was done, the cages were immediately sprayed with ethanol (99%) followed with a thorough rinse using distilled water to remove any residual chemicals on the cages and then a second ethanol spray before the cages were left to air dry. The modified glove and its magnetic frames were soaked in ethanol (99%) in a container, then rinsed with distilled water and a second ethanol rinse, before being left to air dry.

### Single sensillum recording

Single sensillum recording was conducted as described in Liu et al.^48^. Female mosquitoes 4 days after eclosion were anaesthetized (2–3 min on ice) and mounted on a microscope slide (76 × 26 mm)^48^. An antenna was fixed using a double-sided tape to a cover slip resting on a small ball of dental wax to facilitate manipulation. The cover slip was placed at an appropriate angle to the mosquito head. Once mounted, the specimen was placed under a microscope (Eclipse FN1, Japan) and the antenna viewed at a high magnification (1000×). Two tungsten microelectrodes were sharpened in 10% KNO_2_ at 2–10 V. The reference electrode, which was connected to ground, was inserted into the compound eye of the mosquito and the other was connected to the preamplifier (10×, Syntech, Kirchzarten, Germany) and inserted into the shaft of an olfactory sensillum (mainly in 8^th^ to 13^th^ flagellomeres) to complete the electrical circuit to extracellularly record ORN potentials^49^. Controlled manipulation of the electrodes was performed using a micromanipulator (Burleigh PCS-6000, CA). The preamplifier was connected to an analog-to-digital signal converter (IDAC-4, Syntech, Germany), which in turn was connected to a computer for signal recording and visualization in the software AutoSpike v3.1. The activity of co-located ORNs in each sensillum was assessed based on the differences in spike amplitude. The ORN with the large spike amplitude was designated as cell A and cell B with the small spike amplitude^35^. Signals were recorded for 10 s starting 1 s before stimulation, and the action potentials were counted off-line over a 500-ms period before and after stimulation. The spontaneous firing rates observed in the preceding 500 ms were subtracted from the total spike rates observed during the 500-ms stimulation, and counts were recorded in units of spikes s^−1^.

Eighteen compounds (Supplementary Fig. 2) besides pyrethrum from different chemical classes were selected for functional classification of olfactory sensilla with various morphological shapes. Each compound was diluted in dimethyl sulfoxide (DMSO) to a stock solution with a concentration of 100 μg μl^−1^. Subsequently, a series of 10-fold dilutions were made from each of the stock solutions for each compound tested. For each dilution, a 10 μl portion was dispersed onto a filter paper strip (4 × 30 mm), which was then inserted into a Pasteur pipette to create the stimulus cartridge. A sample containing the solvent alone served as control. The airflow across the antennae was maintained constant at a 20 ml s^−1^ throughout the experiment. Purified and humidified air was delivered to the preparation through a glass tube (10-mm inner diameter) perforated by a small hole 10 cm away from the end of the tube, into which the tip of the Pasteur pipette could be inserted. The stimulus was delivered to the sensilla by inserting the tip of the stimulus cartridge into this hole and diverting a portion of the air stream (0.5 l min^−1^) to flow through the stimulus cartridge for 500 ms using a stimulus controller (Syntech, Germany). The distance between the end of the glass tube and the antennae was ≤1 cm. The number of spikes s^−1^ was obtained by averaging the results for each sensillum/compound combination. Recording from each replicate/sensillum was done using different mosquitos.

### Electroantennogram

The electroantennogram procedure followed that described in Pelletier et al.^50^ with minor modifications. Briefly, the head of an adult *Ae. aegypti* female was excised and mounted on an EAG platform equipped with two micromanipulators and a high-impedance AC/DC preamplifier (Syntech, Germany). Chlorinated silver wires in glass capillaries filled with 0.1% KCl and 0.5% polyvinylpyrrolidone (PVP) were used for both reference and recording electrodes. One antenna with the tip cut was accommodated into the recording electrode. Preparation was bathed in a high humidity air stream flowing at 20 ml s^−1^ to which a stimulus pulse of 2 ml s^−1^ was delivered for 500 ms. Any change in antennal deflection induced by the stimuli or control puffs was recoded for 10 s. All compounds were dissolved in paraffin oil to make a stock solution of 10-fold dilution, and decadic dilutions were made. An aliquot (10 μl) of a tested compound was loaded onto a filter paper strip (4 × 30 mm), which was immediately inserted into a Pasteur pipette for evaporation. Solvent (paraffin oil) alone served as control. For each compound, EAG responses of 5 to 11 female mosquitoes were recorded.

### Chemicals

Compounds that were used in electrophysiological recordings and behavioral assays are listed in Supplementary Table 1.

### The empty neuron system

We followed the method of Gonzalez et al.^51^ for heterologous expression of AgORs in the ab3 empty neurons. The Gal4 line (*w; Cyo/Δhalo; Or22a-Gal4*) was kindly provided by John Carlson (Yale Univ.), and 50 UAS-AgOr lines were obtained from the Bloomington *Drosophila* stock center. Flies from each UAS-AgOr line with red eye and curly wings was used to cross with the Gal4 line and progeny with red eye and straight wings were selected for single sensillum recording.

### sgRNA design and production

The procedure for sgRNA synthesis followed the description of Li et al.^52^ with minor modifications. Two guide RNAs (Supplementary Table 2) were designed by searching the sense and antisense strands of the *AaOr31* gene (AAEL013217) for the presence of protospacer-adjacent motifs (PAMs) with the sequence of NGG using the Chopchop online tool (http://chopchop.cbu.uib.no/). Linear double-stranded DNA templates for all sgRNAs were generated by template-free polymerase chain reaction (PCR) using NEB Q5 high-fidelity DNA polymerase (catalog # M0491S) and a sense primer AaOR31crisprF-1 or AaOR31crisprF-2 paired with an antisense primer AaOR31crisprR (Supplementary Table 2). PCR reactions were heated to 98 °C for 30 s, followed by 35 cycles of 98 °C for 10 s, 58 °C for 10 s, and 72 °C for 10 s, then 72 °C for 2 min. PCR products were purified using Promega Wizard@SV Gel and PCR Clean-up System (catalog #A9281). Following PCR, sgRNAs were synthesized using the Ambion Megascript T7 in vitro transcription kit (catalog # AM1334, Life Technologies) according to the manufacturer’s protocol using 300 ng of purified DNA template. Following in vitro transcription, the sgRNAs were purified using the MegaClear Kit (catalog #AM1908, Life Technologies) and diluted to 1000 ng μl^−1^ in nuclease-free water and stored in aliquots at −80 °C. Recombinant Cas9 protein from *Streptococcus pyogenes* was obtained commercially (CP01, PNA Bio Inc) and diluted to 1000 ng l^−1^ in nuclease-free water and stored in aliquots at −80 °C.

### CRISPR mediated microinjections

Embryonic collection and CRISPR microinjections were performed following the procedure described by Li et al.^52^. Briefly, Rockefeller mosquitoes were blood-fed 5 days before egg collection. An ovicup filled with ddH_2_O and lined with filter paper was placed into a cage and female mosquitoes were allowed to lay eggs in the ovicup in the dark. After 15–30 min, the ovicup was taken out and unmelanized eggs were transferred onto a glass slide. The eggs were quickly aligned on a wet piece of filter paper. Aluminosilicate needles were pulled on a Sutter P-1000 needle puller and beveled using a Sutter BV-10 beveler. An Eppendorf Femotojet was used for power injections under a compound microscope at 100× magnification. About 50 eggs were injected each time immediately after fresh eggs were collected. The concentration of components used in the study was as follows; Cas9 protein at 300 ng μl^−1^, each sgRNA at 40 ng μl^−1^. After injection, eggs were placed in a cup filled with water and allowed to hatch and developed into adults. To identify mutants, we performed genotyping at each generation of mosquitoes after injection by cutting one hind leg off for genomic DNA isolation. Genomic DNA was extracted using the DNeasy blood & tissue kit (QIAGEN) following the manufacturer’s protocol. Target loci were amplified by PCR using the primers AaOR31F (5’-ATTGGCATGCGCTACTTTTATT-3’) and AaOR31R (5’-ATAACATCCTTTAGCCAGTGCC-3’) (also in Supplementary Table 2). PCR products were gel purified and sent directly for Sanger sequencing using the forward primer used in PCR reactions.

### Data analysis, statistics and experimental repeats

All statistical analysis was done using Prism 5 (GraphPad Software). Data are presented as mean ± s.e.m. Unpaired Student’s *t*-tests was used to compare two sets of data. If the data did not meet the normality or equality of the variance assumptions needed for Student’s *t*-tests, the equivalent Mann–Whitney Rank Sum test was used instead. The significance for all the tests was set to a *P-*value <0.05. Each hand-in-cage experiment and SSR recording were repeated by two or more researchers.

### Reporting summary

Further information on research design is available in the Nature Research Reporting Summary linked to this article.

## Supplementary information

Supplementary Information

Reporting Summary

## Data Availability

The data that support all experimental findings of this study are available within the paper and its Supplementary Information files. Raw data necessary to reproduce all statistical analyses and results in the paper as well as P values for all figures are provided in the source data file. Source data are provided with this paper. Vectorbase (www.vectorbase.org) and the NCBI website (http://www.ncbi.nlm.nih.gov) were used.
