# Supplementary material for: A dual-target molecular mechanism of pyrethrum repellency against mosquitoes
Source: Nat Commun. 2021 May 5;12:2553. doi: 10.1038/s41467-021-22847-0 (PMC8099882; doi:10.1038/s41467-021-22847-0)
Supplement: Supplementary file 1 — Supplementary Information [file 41467_2021_22847_MOESM1_ESM.pdf]

Supplementary Information for:

A dual-target molecular mechanism of pyrethrum repellency against mosquitoes

Feng Liu, Qiang Wang, Peng Xu, Felipe Andreazza, Wilson R. Valbon, Elizabeth Bandason, Mengli Chen, Ru Yan, Bo Feng, Leticia Smith, Jeffrey G. Scott, Genki Takamatsu, Makoto Ihara, Kazuhiko Matsuda, James Klimavicz, Joel Coats, Eugenio E Oliveira, Yuzhe Du, Ke Dong*

*Correspondence to: ke.dong@duke.edu

This file contains:

Supplementary Figures: 1-13

Supplementary Tables: 1-3

Supplementary Notes: 1

Supplementary References: 1-2

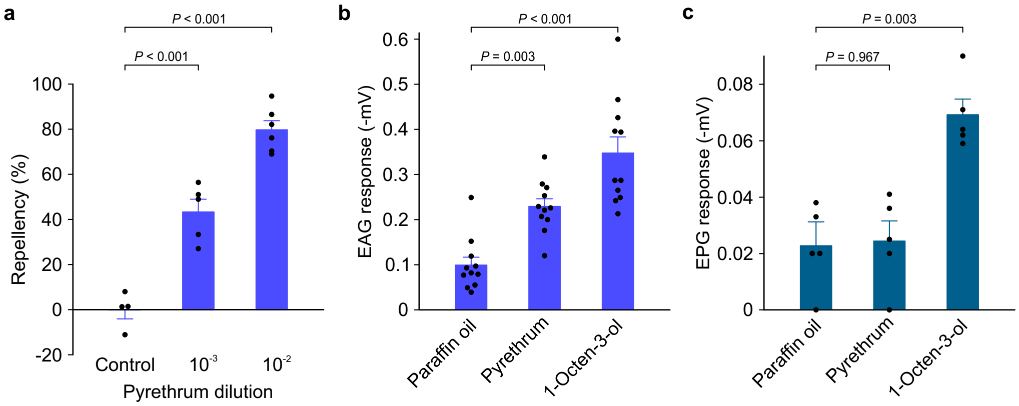

Supplementary Figure 1. **a**, Pyrethrum repellency against *An. gambiae* Kisumu mosquitoes (n = 4 cage for control; n = 5 cages at the 10^{-3} dilution (v v^{-1}); n = 6 cages at the 10^{-2} dilution (v v^{-1}) from two batches of mosquitoes; t = 5.98, df = 7, $P < 0.001$ for control *vs.* pyrethrum at 10^{-3} and t = 13.36, df = 8, $P < 0.001$ for control *vs.* pyrethrum at 10^{-2}). **b**, EAG responses of *An. gambiae* Kisumu mosquitoes to pyrethrum and 1-octen-3-ol (One-way RM ANOVA with Dunnett's test, $F_{(2,20)}$ = 25.39, $P < 0.001$, n = 11 antennae). **c**, No response from maxillary palps to pyrethrum in Rockefeller mosquitoes. 1-octen-3-ol is used as a positive control (One-way RM ANOVA with Dunnett's test, $F_{(2,8)}$ = 13.52, P = 0.003, n = 5 maxillary palps). The concentration of pyrethrum used was 10^{-2} dilution (v v^{-1}). Data are plotted as mean ± s.e.m. and dots denote the value of each repeat.

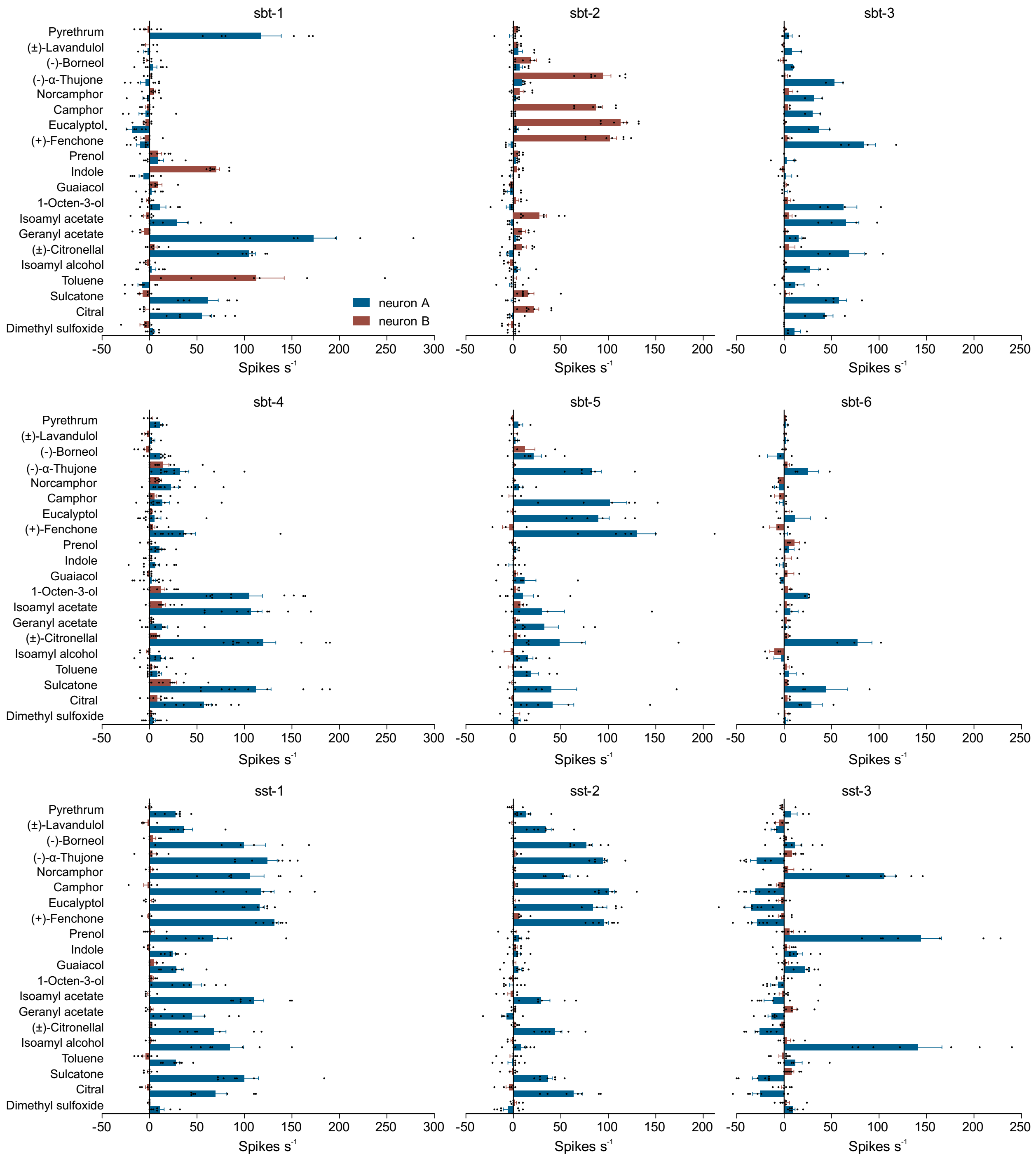

Supplementary Figure 2. The response profiles of three short sharp-tipped (sst) and six short blunt-tipped (sbt) sensilla identified in the single sensillum recording in Rockefeller mosquitoes. Value of *n* for each compound is presented in Supplementary Table 3. Data are plotted as mean ± s.e.m. and dots denote the value of each repeat.

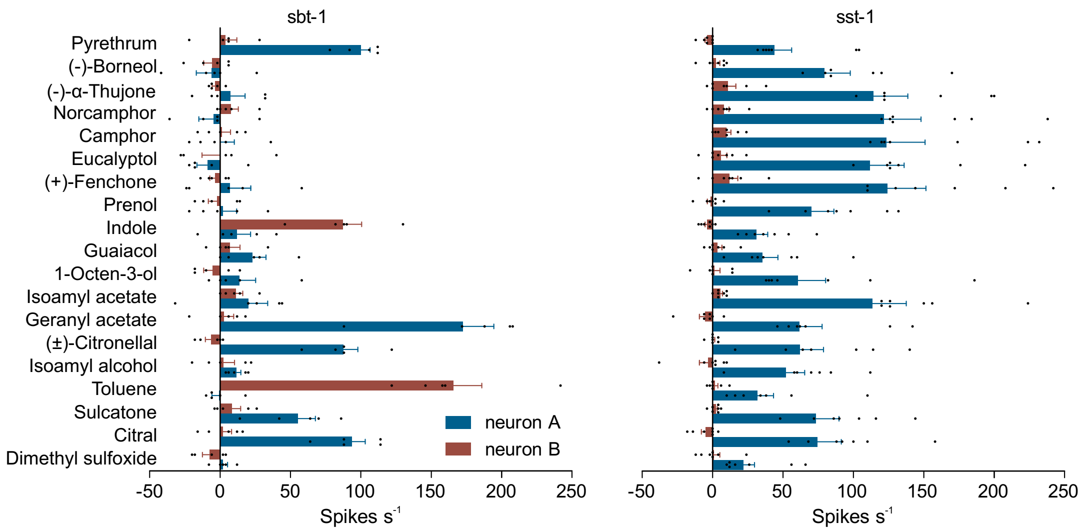

Supplementary Figure 3. The response profiles of short sharp-tipped (sbt)-1 and short sharp-tipped (sst)-1 sensilla from Orlando mosquitoes (*n* = 5 sensilla for sbt-1 and *n* = 7 sensilla for sst-1). Data are plotted as mean ± s.e.m. and dots denote the value of each repeat.

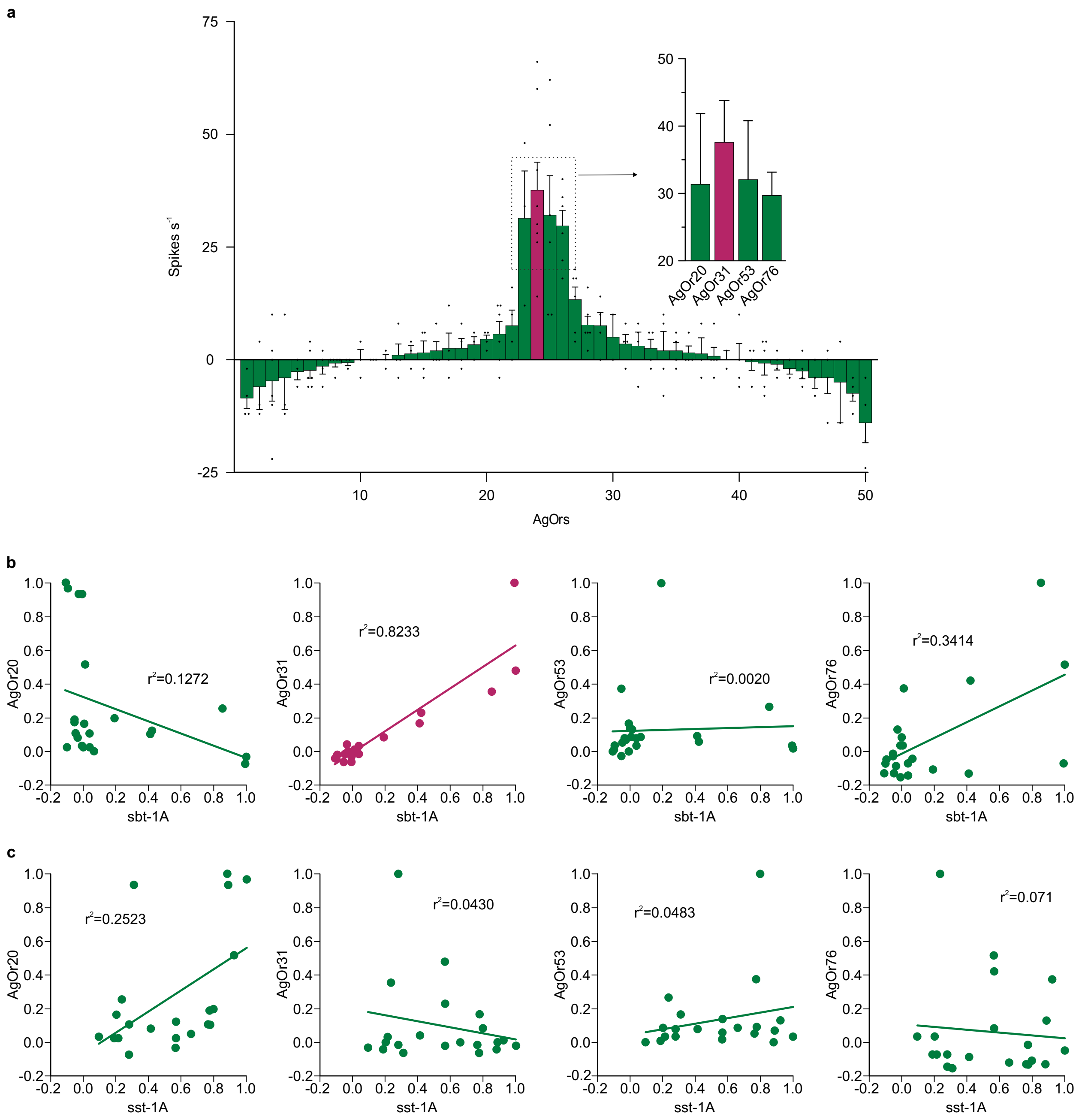

Supplementary Figure 4. a, Responses of ab3A neurons expressing each of 50 AgOrs to pyrethrum. Four AgOrs (AgOr31, AgOr20, AgOr53 and AgOr76) were activated significantly (Δ spikes $s^{-1} > 20$) by pyrethrum at the 10^{-2} dilution (v v^{-1}) (*n* = 3 ab3A neurons for AgOr20; *n* = 6 for AgOr53 and AgOr76; *n* = 8 for AgOr31). **b** and **c**, No correlation between the response profiles of AgOr20, AgOr53 or AgOr76 with the profiles of sbt-1 (**b**) or sst-1 (**c**) sensilla to 20 odorants. Data are plotted as mean ± s.e.m. and dots denote the value of each repeat.

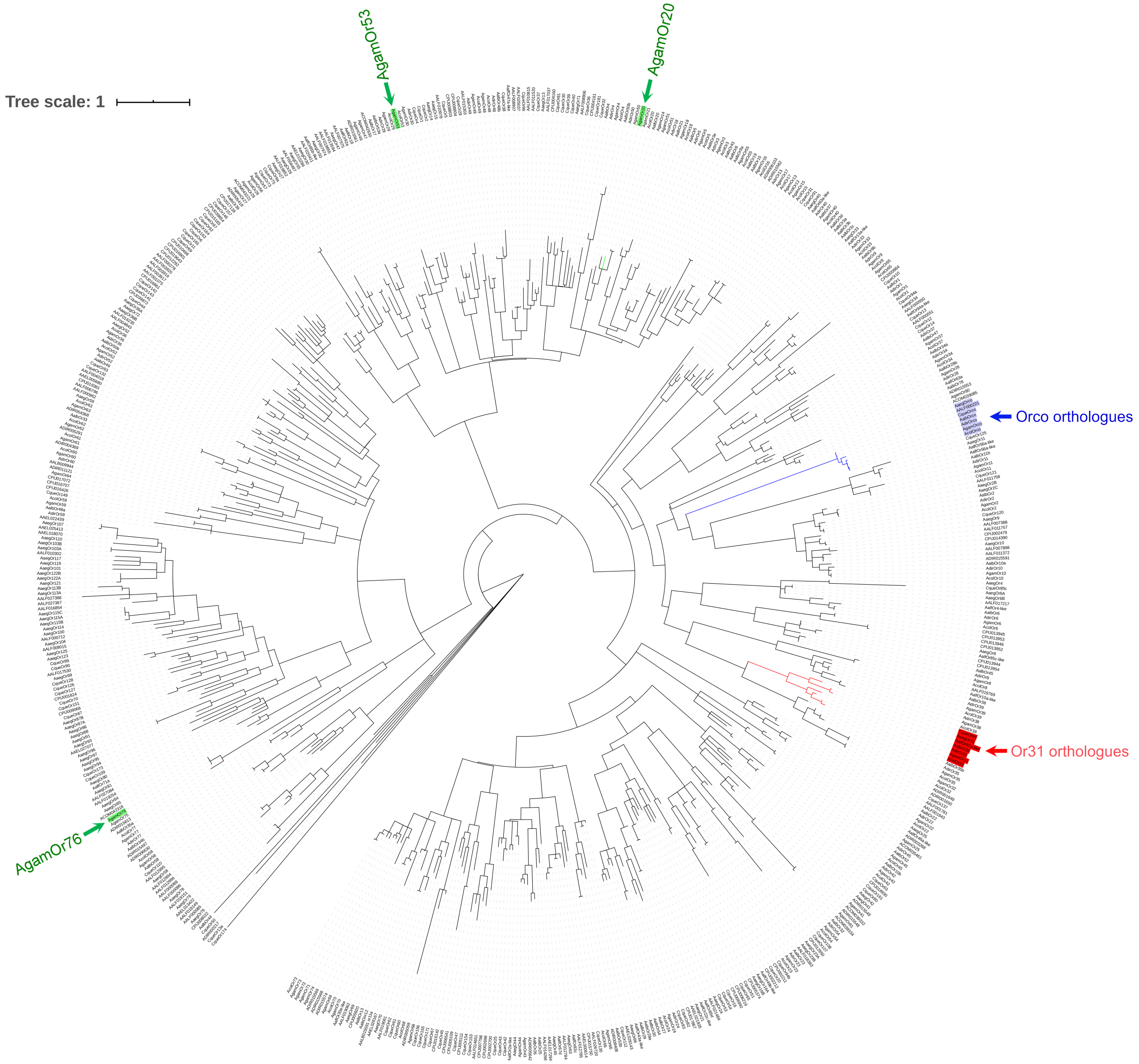

Supplementary Figure 5. Phylogenetic relationship of the odorant receptors of seven mosquito species. Sequences encoding odorant receptors (Ors) from *Aedes aegypti*, *Aedes albopictus, Anopheles gambiae*, *Anopheles coluzzii*, *Anopheles dirus*, *Anopheles albimanus*, and *Culex quinquefaciatus* were retrieved from Vectorbase (www.vectorbase.org). Additional sequences of *Ae. albopictus* Ors were retrieved from the NCBI website (http://www.ncbi.nlm.nih.gov). ClustalW alignment of the amino acid sequences was conducted with Bioedit v7.2 (https://bioedit.software.informer.com/7.2/). The maximum likelihood phylogenetic tree was constructed with 500 bootstrap replicates using MEGA 6 (https://www.megasoftware.net/) based on amino acid sequence alignment and was further refined with iTOL v5 (https://itol.embl.de/). Distinct branches consisting of Or31 and putative orthologues (red color) and Orco and its putative orthologues (blue color) are indicated. In addition, three anopheles-specific pyrethrum-responsive AgOrs (i.e., AgamOrs, green color) are also indicated.

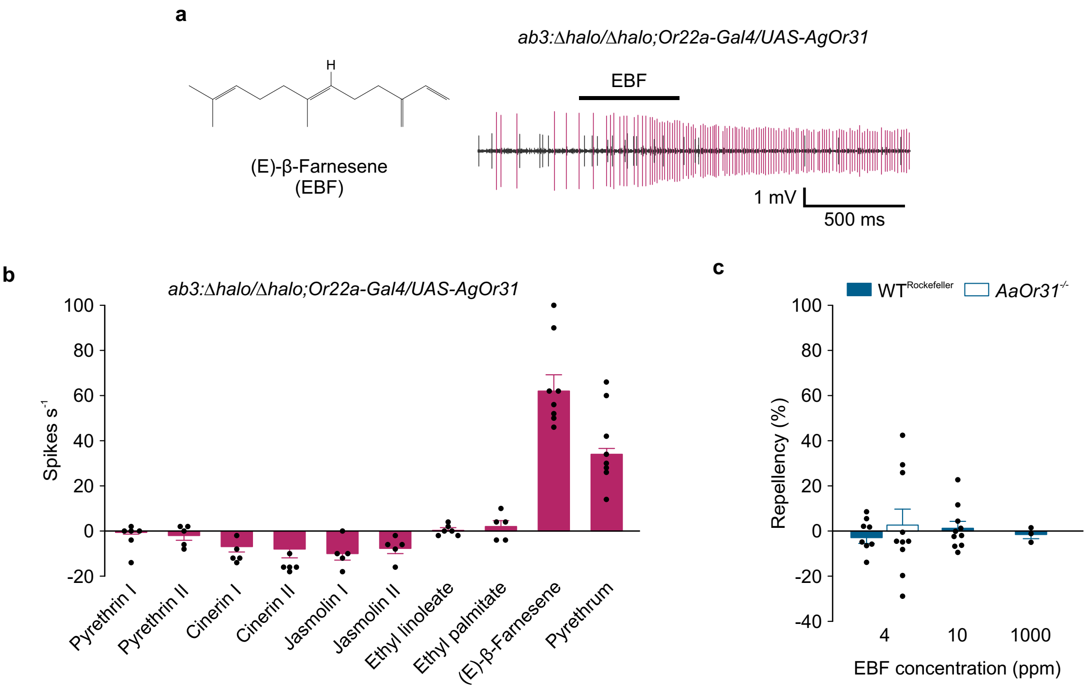

Supplementary Figure 6. a, (E)-β-farnesene (EBF) activated *Drosophila* ab3A empty neurons expressing AgOr31 (from *n* = 5 sensilla). **b**, None of the six insecticidal esters, ethyl palmitate or ethyl linoleate activated AgOr31 in *Drosophila* ab3A empty neurons (*n* = 5 sensilla). **c**, EBF elicited no repellency at the 10^{-3} dilution (i.e., 1000 ppm or 11.4 μg cm^{-2}) or lower concentrations. Rockefeller: *n* = 8 cages for 4 ppm, *n* = 10 cages for 10 ppm, *n* = 3 cages for 1000 ppm, from 2 batches of mosquitoes; *AaOR31*$^{-/-}$: *n* = 10 cages for 4 ppm from 2 batches of mosquitoes. Data are plotted as mean ± s.e.m. and dots denote the value of each repeat.

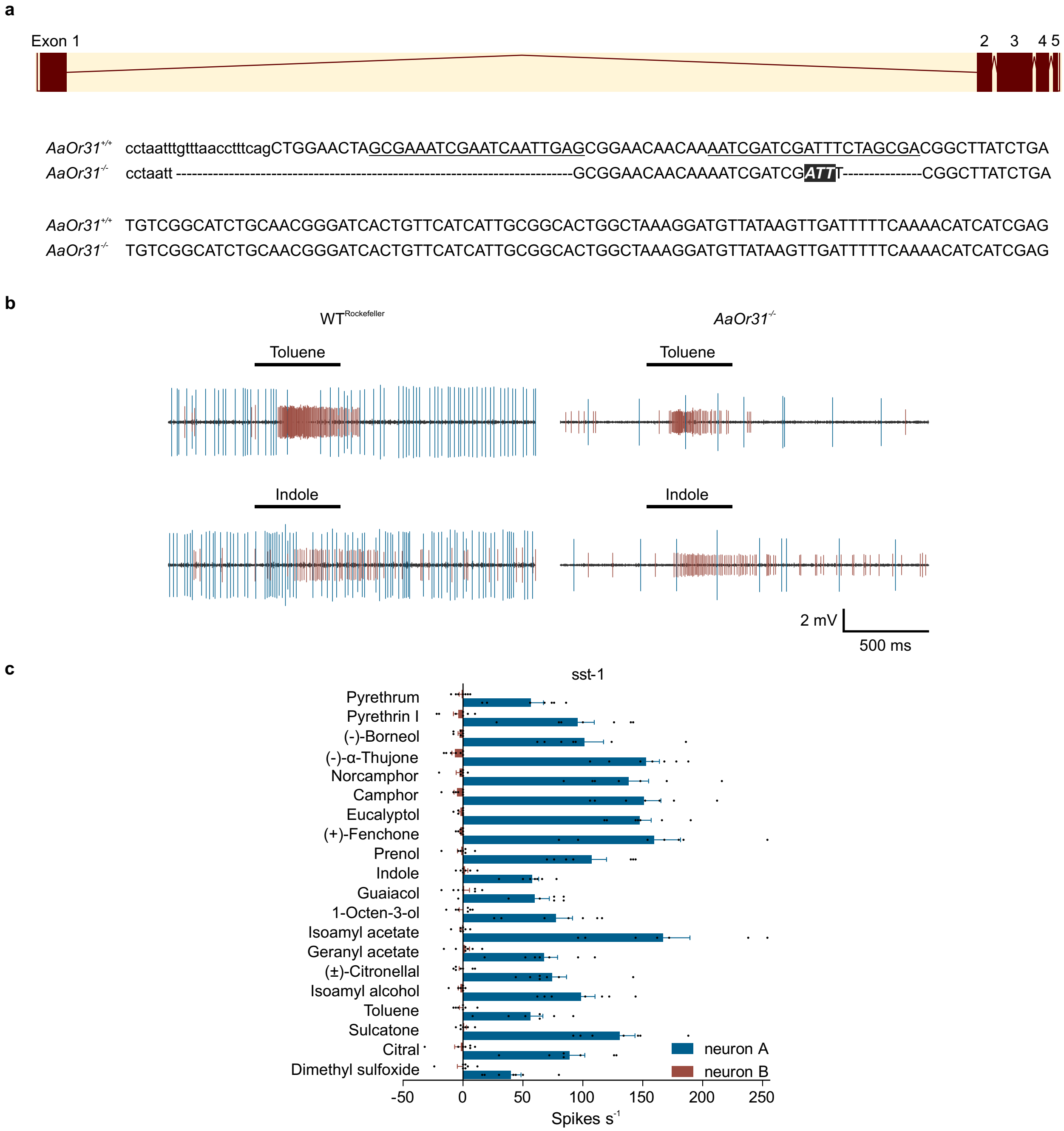

Supplementary Figure 7. a, Alignment of exon 2 (upper case) and upstream intron (lower case) sequences of *AaOr31* between Rockefeller and *AaOr31*$^{-/-}$ strains. Sequences underlined were used in designing two single guide RNAs for CRISPR-Cas9; Two deletions in exon 2 of *AaOr31*$^{-/-}$ are indicated in dashed lines; the premature stop codon caused by frameshift in *AaOr31* after the deletion is highlighted in black shade. **b**, Response of short blunt-tipped (sbt)-1B neurons in *AaOr31*$^{-/-}$ mosquitoes to indole (from n = 8 sensilla) and toluene (from n = 8 sensilla) remained intact. **c**, Knockout of *AaOr31* did not alter short sharp-tipped (sst)-1A response to pyrethrum, pyrethrin I (P-I) or other odorants examined (from n = 7 sensilla). Data are plotted as mean ± s.e.m. and dots denote the value of each repeat.

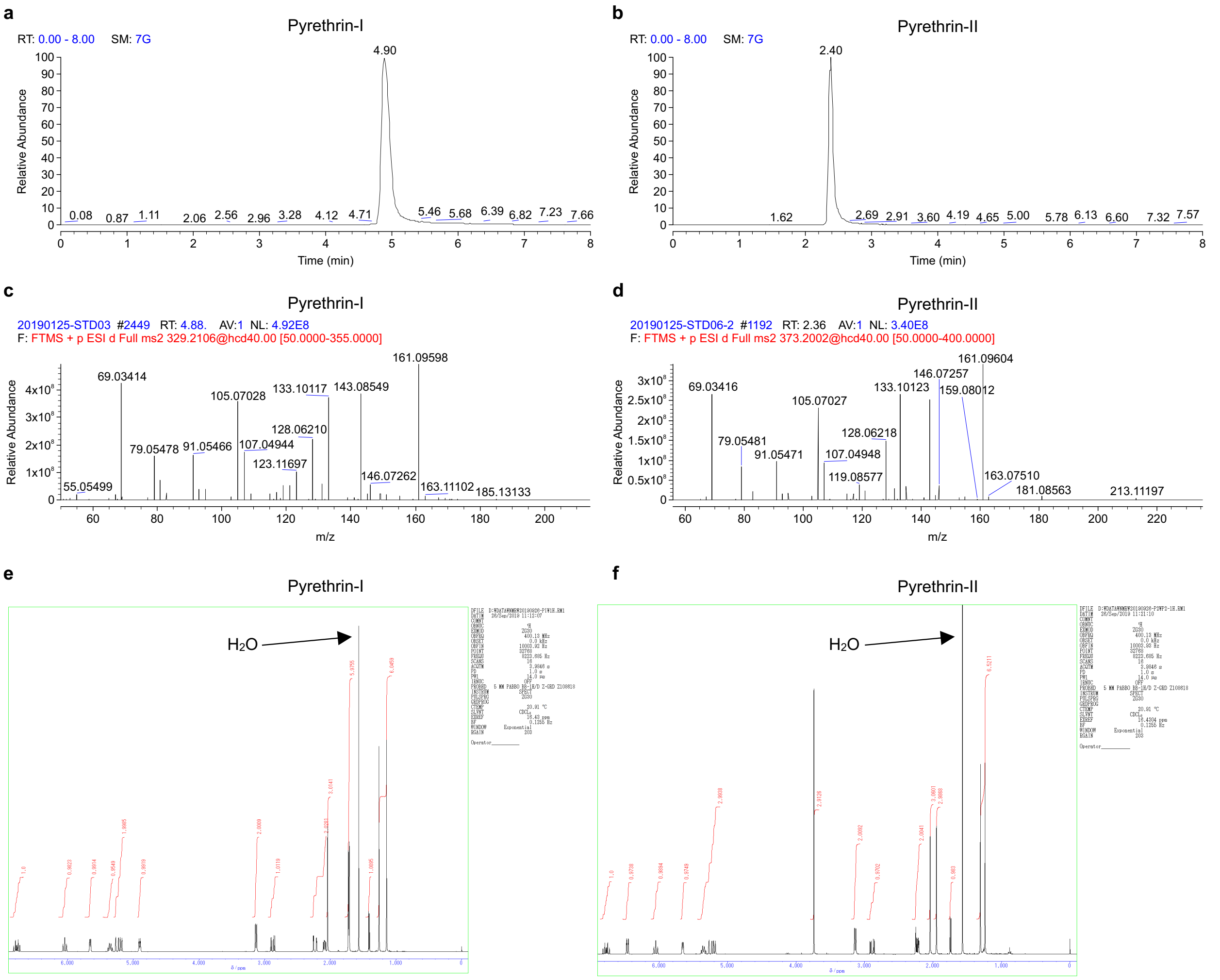

Supplementary Figure 8. Purities of pyrethrins I and II used in this study were validated by high resolution liquid chromatography tandem mass spectrometry (LC-MS) and nuclear magnetic resonance spectroscopy (NMR). **a** and **b**, LC chromatograms of pyrethrin I (**a**) and pyrethrin II (**b**). **c** and **d**, MS spectra of pyrethrin I (**c**) and pyrethrin II (**d**). For liquid chromatography, chromatographic column: Acquity UPLC BEH C18 (2.1 mm × 150 mm, 1.7 µm, Waters, USA), mobile phase: 0.1% formic acid-aqueous solution (mobile phase A) and 0.1% formic acid-acetonitrile solution (mobile phase B), run time: 10 min, flow rate: 0.3 ml min^{-1}, column temperature: 35 °C, isocratic elution mode: 80%B. For Mass spectrometry, Ion source: HESI+, Spray voltage: 3.8 kV, Sheath gas flow rate: 40, Aux gas flow rate: 10, Capillary temperature: 320 °C, S-lens RF level: 55, Aux gas heater temperature: 350°C, Data acquisition mode: full MS dd-ms^{-2}(top N), full MS mode: Resolution: 70000, AGC target: 1e6, maximum IT: 200 Ms, Scan range: 50 to 750 m z^{-1}, spectrum data type: profile; dd MS^2 mode: Resolution: 17500, AGC target: 1e5, maximum IT: 50 Ms, Top N: 5, Isolation window: 1.6 m z^{-1}, (N)ce/stepped (N) CE: 20, 40, 60, spectrum data type: centroid; dd settings with Minimum AGC target: 8.00e3, Dynamic exclusion: 10 s. **e** and **f**, ^{1}H-NMR spectra of pyrethrin I (**e**) and pyrethrin II (**f**). ^{1}H-NMR spectra were recorded in $CDCl_3$ using an AV400M spectrometer (Bruker).

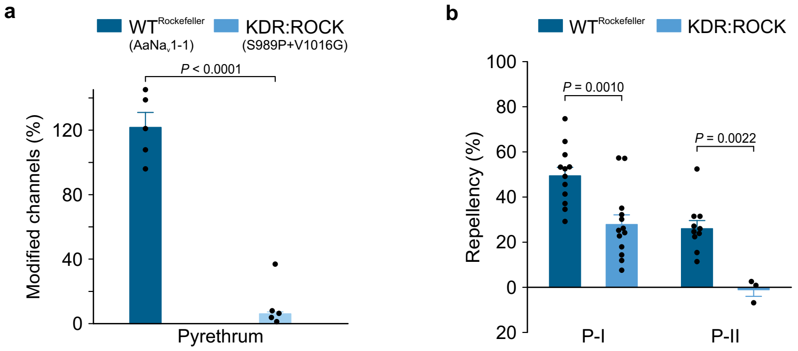

Supplementary Figure 9. **a**, Two kdr mutations, S989P and V1016G, in the mosquito sodium channel ($AaNa_v1$-1) conferred $AaNa_v1$-1 channels resistance to pyrethrum (t = 9.8, df = 8, $P < 0.0001$, n = 5 oocytes for Rockefeller $AaNa_v1$-1 and n = 5 oocytes for KDR:ROCK $AaNa_v1$-1). $AaNa_v1$-1 wild type and the mutant channel carrying the double mutations were expressed in *Xenopus* oocytes and channel sensitivity to pyrethrum was examined using the two-electrode voltage clamp technique[1] with pCLAMP v10. The effect of pyrethrum was measured 10 minutes after its application. Pyrethrum-induced tail currents were recorded during a 100-pulse train of 5 milliseconds step depolarizations from -120 to -10 mV with 5 milliseconds inter-pulse intervals[1]. The percentage of pyrethrum-modified channels was calculated using the following equation: $M = [I_{tail} / (E_h - E_{Na})] / [I_{Na} / (E_t - E_{Na})] \times 100$; where I_{tail} is the maximal tail current amplitude, E_h is the potential to which the membrane is repolarized, E_{Na} is the reversal potential for sodium currents determined from the current-voltage curve, I_{Na} is the amplitude of the peak current during depolarization before pyrethrum exposure, and E_t is the potential of the step depolarization. **b**, Reduced pyrethrin I and II (10^{-3} v v^{-1}) repellency in KDR:ROCK mosquitoes compared to that in Rockefeller (wild-type) mosquitoes (P-I: t = 3.78, df = 23, P = 0.0010, n = 12 cages for Rockefeller and n = 13 cages for KDR:ROCK from 3 batches of mosquitoes; P-II: t = 3.97, df = 11, P = 0.0022, n = 10 cages for Rockefeller and n = 3 cages for KDR:ROCK from 2 batches of mosquitoes). Two-tailed unpaired student's t-test was used to compare each of two sets of data. Data are plotted as mean ± s.e.m. and dots denote the value of each repeat.

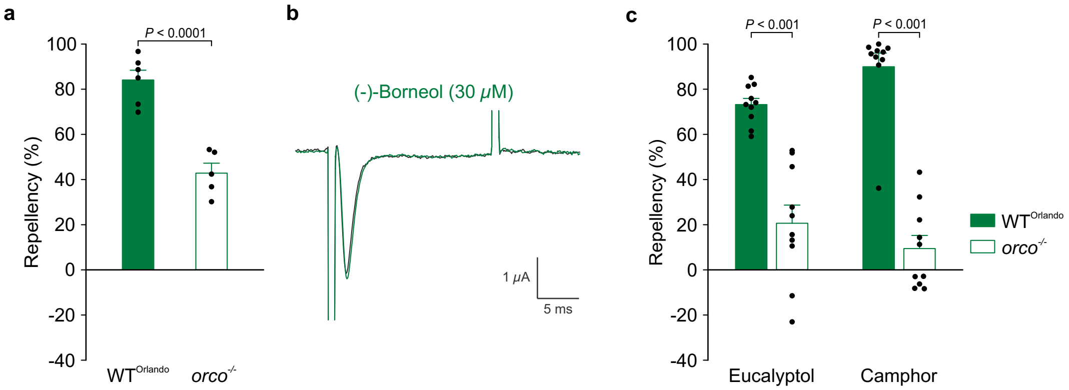

Supplementary Figure 10. a, Repellency by (-)-borneol (10^{-2} v v^{-1}) is Orco-mediated (t = 6.64, df = 9, P < 0.0001, n = 6 cages for *Orlando (wild-type)* and n = 5 cages for *orco*$^{-/-}$ from 3 batches of mosquitoes). **b**, Sodium current recording traces from $AaNa_v1$-1 channels expressed in *Xenopus* oocytes in response to 20 ms step-depolarization before and after exposed (-)-borneol (from n = 5 oocytes from two separated batches). (-)-borneol had no effects on peak current and channel gating. **c**, Repellency by eucalyptol and camphor (10^{-2} v v^{-1}) is Orco-mediated (eucalyptol: U = 0; P < 0.001, camphor: U = 1.0; P < 0.001, n = 10 cages for each mosquito strain/compound, from two batches of mosquitoes). Two-tailed unpaired student's t-test or two-tailed Mann-Whitney Rank Sum test was used to compare each of two sets of data. Data are plotted as mean ± s.e.m. and dots denote the value of each repeat.

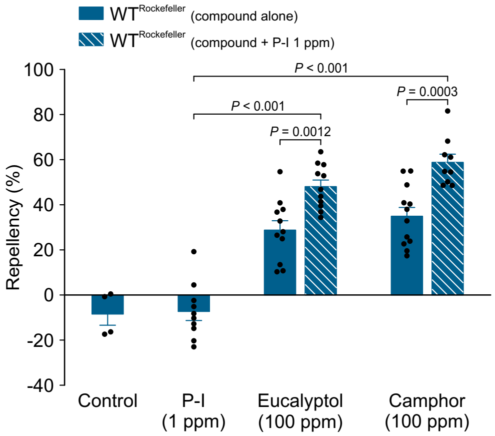

Supplementary Figure 11. Pyrethrin I (P-I) enhanced Orco-dependent repellency by eucalyptol and camphor in Rockefeller (wild-type) mosquitoes. (Two-tailed unpaired student's *t*-test: P-I *vs.* P-I + eucalyptol: $t = 11.46$, $df = 19$; $P < 0.001$, P-I *vs.* P-I + camphor: $t = 12.33$, $df = 17$, $P < 0.001$; eucalyptol *vs.* P-I + eucalyptol: $t = 3.78$, $df = 20$, $P = 0.0012$; camphor *vs.* P-I + camphor: $t = 4.37$, $df = 19$; $P = 0.0003$; $n = 4$ cages for control, $n = 9$ cages for camphor + P-I, $n = 10$ cages for P-I, $n = 11$ cages for eucalyptol and eucalyptol + P-I, and $n = 12$ cages for camphor, from 2 batches of mosquitoes). Data are plotted as mean ± s.e.m. and dots denote the value of each repeat.

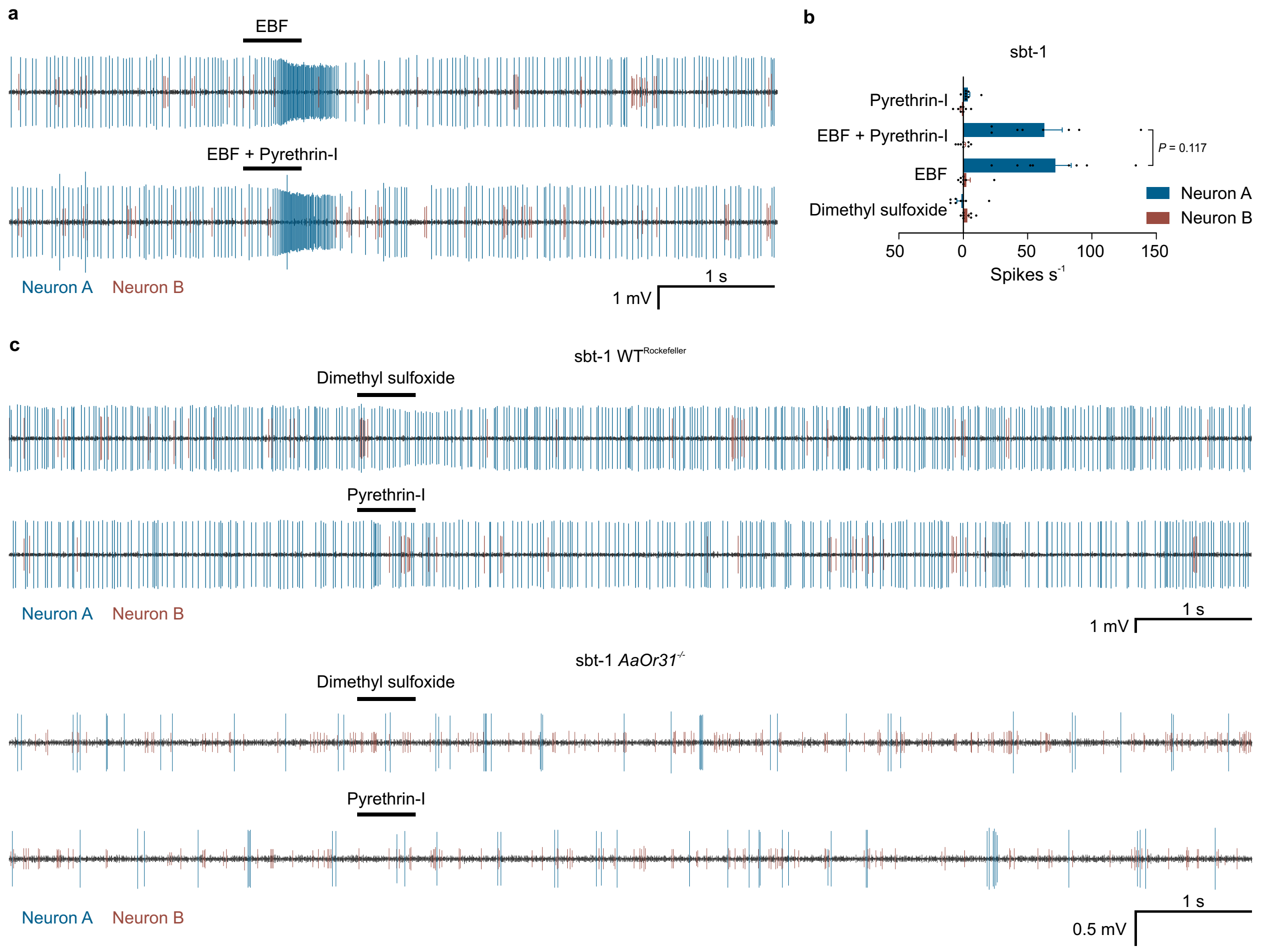

Supplementary Figure 12. a, Representative single sensillum recording (SSR) traces (from *n* = 8 sensilla) evoked by (E)-β-farnesene (EBF) with or without co-application of pyrethrin I in short blunt-tipped (sbt)-1 sensilla in Rockefeller (wild-type) mosquitoes. **b**, Co-application of pyrethrin I and EBF did not affect the EBF-evoked activity (t = 1.79, df = 7, P = 0.117, n = 8 sensilla). **c**, Pyrethrin I did not affect the spontaneous firing of sbt-1 neurons from either Rockefeller or *AaOr31*$^{-/-}$ mosquitoes (from *n* = 7 sensilla for each mosquito strain). Two-tailed paired Student's *t*-test was used to compare the two sets of data. Data are plotted as mean ± s.e.m. and dots denote the value of each repeat.

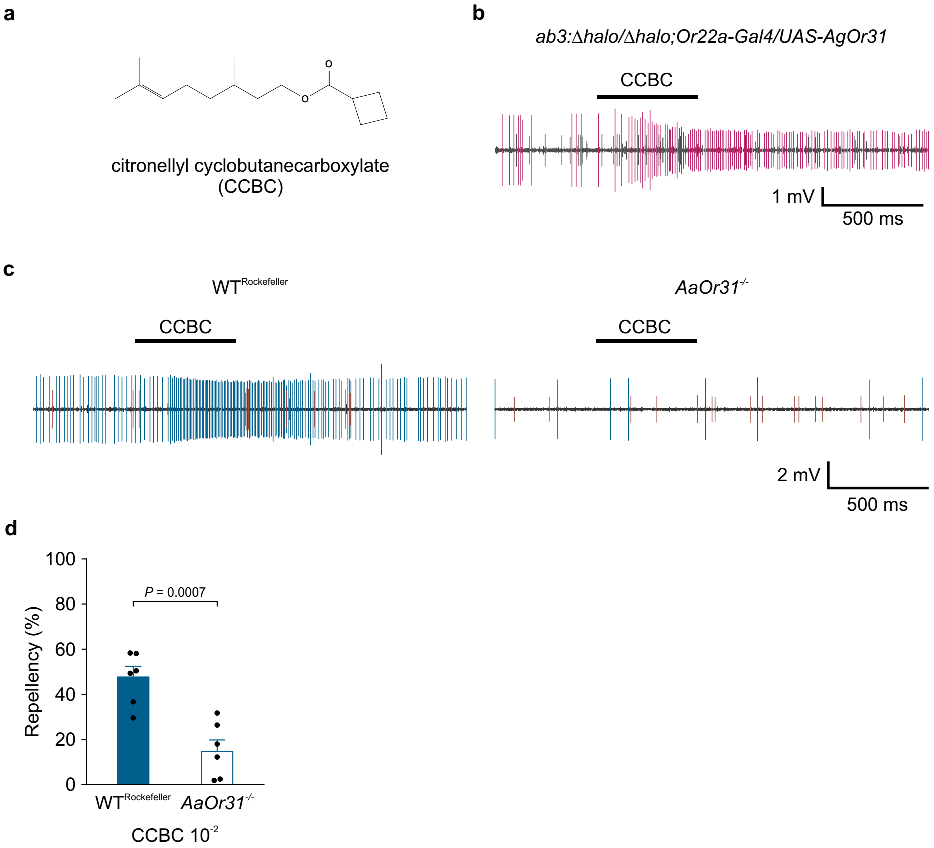

Supplementary Figure 13. AaOr31-mediated repellency by a synthetic mosquito repellent citronellyl cyclobutanecarboxylate (CCBC). **a**, CCBC chemical structure: Colorless oil. ^{1}H NMR (400 MHz, CDCl3) δ 5.08 (thept, *J* = 7.1, 1.4 Hz, 1H), 4.17 – 4.03 (m, 2H), 3.11 (pd, *J* = 8.5, 1.1 Hz, 1H), 2.36 – 2.23 (m, 2H), 2.22 – 2.11 (m, 1H), 2.07 – 1.81 (m, 4H), 1.71 – 1.65 (m, 1H), 1.67 (d, *J* = 1.5 Hz, 3H), 1.60 (d, *J* = 1.3 Hz, 3H), 1.57 – 1.49 (m, 1H), 1.48 – 1.39 (m, 1H), 1.34 (dddd, *J* = 13.3, 9.3, 6.5, 5.4 Hz, 1H), 1.18 (dddd, *J* = 13.5, 9.2, 7.6, 6.1 Hz, 1H), 0.91 (d, *J* = 6.6 Hz, 3H). ^{13}C NMR (101 MHz, CDCl3) δ 175.7, 131.5, 124.7, 62.9, 38.3, 37.1, 35.6, 29.6, 25.8, 25.5, 25.41, 25.39, 19.6, 18.5, 17.8. **b**, AgOr31 expressed in *Drosophila* empty neurons was activated by CCBC at the 10^{-2} dilution (v v^{-1}) (from *n* = 6 sensilla). **c**, CCBC activates sbt-1A neurons in Rockefeller (wild-type) mosquitoes (from *n* = 10 sensilla), but not in *AaOr31*$^{-/-}$ mosquitoes (from *n* = 2 sensilla). **d**, CCBC repellency was significantly reduced in *AaOr31*$^{-/-}$ mosquitoes (t = 4.77, df = 10, P = 0.0007, n = 6 cages for Rockefeller and n = 6 cages for *AaOr31*$^{-/-}$ from 2 batches of mosquitoes). Two-tailed unpaired student's *t*-test was used to compare the two sets of data. Data are plotted as mean ± s.e.m. and dots denote the value of each repeat.

Supplementary Table 1. Compounds used in both electrophysiological recordings and behavioral assays.

Chemical name	CAS	Purity	Source
Citral	5392-40-5	95.7%	Sigma
Sulcatone	110-93-0	99.2%	Sigma
Toluene	108-88-3	100%	J.T. Baker
Isoamyl alcohol	123-51-3	99.5%	Sigma
(±)-citronellal	106-23-0	99.%	Sigma
Geranyl acetate	105-87-3	98.3%	Sigma
Isoamyl acetate	123-92-2	99.43%	Sigma
1-octen-3-ol	3391-86-4	99.4%	Sigma
Guaiacol	90-05-1	99.4%	Sigma
Indole	120-72-9	99.6%	Sigma
Prenol	556-82-1	99.6%	Sigma
(+)-Fenchone	4695-62-9	99.7%	Sigma
Eucalyptol	470-82-6	99.7%	Sigma
Camphor	76-22-2	95.7%	Sigma
Norcamphor	497-38-1	99.1%	Sigma
(-)-α-thujone	546-80-5	98.4%	Sigma
(-)-Borneol	464-45-9	99.7%	Sigma
Lavandulol	58460-27-1	96.04%	Indofine Chem. Company
Pyrethrum extract	8003-34-7	73.1% (sum of pyrethrins)	Sigma
(E)-β-Farnesene	18794-84-8	98.5	Sigma
Ethyl linoleate	544-35-4	≧99%	Sigma
Ethyl palmitate	628-97-7	≧99%	Sigma
Dimethyl sulfoxide	67-68-5	100%	Sigma
Paraffin oil	8012-95-1	-[†]	Sigma
Acetone	67-64-1	99.98%	Sigma
Citronellyl cyclobutanecarboxylate (CCBC)	-[††]	>98%	Synthesized
Pyrethrin I	121-21-1	≧99%	Purified
Pyrethrin II	121-29-9	≧99%	Purified
Cinerin I	97-12-1	≧99%	Purified
Cinerin II	204-454-2	≧99%	Purified
Jasmolin I	4466-14-2	≧99%	Purified
Jasmolin II	1172-63-0	≧99%	Purified

[†] mixture of density = 0.864. [††] a recently-synthesized experimental compound. The method of synthesis is described in Supplementary Notes 1 below.

Supplementary Table 2. Primers used in CRISPR/Cas9-mediated mutagenesis and genotyping.

Oligonucleotide name	Sequence#
AaOR31crisprF-1	5’GAAATTAATACGACTCACTATAGGAATCGATCGATTTCTAGCGAGTTTTAGAGCTAGAAATAGC3’
AaOR31crisprF-2	5’GAAATTAATACGACTCACTATAGGCGAAATCGAATCAATTGAGGTTTTAGAGCTAGAAATAGC3’
AaOR31crisprR	5’AAAAGCACCGACTCGGTGCCACTTTTTCAAGTTGATAACGGACTAGCCTTATTTTAACTTGCTATTTCTAGCTCTAAAAC3’
AaOR31ExamF	5’ATTGGCATGCGCTACTTTTATT3’
AaOR31ExamR	5’ATAACATCCTTTAGCCAGTGCC3’

#: Underlined sequences were used for guide RNA design.

Supplementary Table 3. Specific number of replicates (insects) for Supplementary Figure 2.

Chemical	Sensillum and neuron																	
	sbt-1		sbt-2		sbt-3		sbt-4		sbt-5		sbt-6		sst-1		sst-2		sst-3	
	A	B	A	B	A	B	A	B	A	B	A	B	A	B	A	B	A	B
Pyrethrum	6	7	7	7	4	3	5	4	4	2	2	2	7	7	7	7	5	6
(±)-Lavandulol	4	4	7	6	2	3	5	4	4	2	2	2	6	3	7	4	4	5
(-)-Borneol	7	7	7	7	2	3	10	9	6	4	3	3	6	5	7	6	7	7
(-)-αThujone	7	6	7	7	2	3	10	9	6	4	3	3	7	6	7	6	7	7
Norcamphor	7	7	7	7	2	3	10	9	6	4	3	3	7	6	7	6	7	7
Camphor	7	7	7	7	2	3	10	9	6	4	3	3	7	6	7	6	7	7
Eucalyptol	6	7	7	7	2	3	10	9	6	4	3	3	7	6	7	6	7	7
(+)-Fenchone	6	7	7	7	4	3	10	9	6	4	3	3	7	6	7	7	7	7
Prenol	7	7	7	7	3	3	10	9	6	4	3	3	7	6	7	7	7	7
Indole	7	7	7	7	3	3	10	9	6	4	3	3	7	6	7	7	7	7
Guaiacol	7	6	7	7	3	3	10	9	6	4	3	3	6	5	7	7	7	7
1-Octen-3-ol	7	7	7	7	4	3	10	9	6	4	3	3	7	6	7	7	7	7
Isoamyl acetate	7	7	7	7	4	3	10	9	6	4	3	3	7	6	7	7	7	7
Geranyl acetate	7	7	7	7	4	3	10	9	6	4	3	3	7	7	7	7	7	7
(±)-Citronellal	7	7	7	7	4	3	10	8	6	4	3	3	7	7	7	7	7	7
Isoamyl alcohol	7	7	7	7	4	3	10	9	6	4	3	3	7	7	7	7	7	7
Toluene	7	7	7	7	4	3	10	9	6	4	3	3	7	7	7	7	7	7
Sulcatone	6	7	7	7	4	3	10	9	6	4	3	3	7	7	7	7	7	7
Citral	7	7	7	7	4	3	10	9	6	4	3	3	7	7	7	7	7	7
Dimethyl sulfoxide	7	7	7	7	3	3	10	9	6	4	3	3	7	6	7	7	7	7

Supplementary Notes 1. Synthesis of Citronellyl cyclobutanecarboxylate (CCBC)

This repellent ester was synthesized using the Steglich esterification[2]. Specifically, for citronellyl cyclobutanecarboxylate, citronellol (781 mg, 5 mmol) was dissolved in dichloromethane (DCM, 25 ml), and cyclobutanecarboxylic acid (6 mmol) and DMAP (61.1 mg, 0.5 mmol) were added. The solution was cooled to 0 °C, and DCC (5.25 mmol, 1.08 g) was added portion-wise. After the addition of DCC, the reaction was stirred at 0 °C for 15 minutes, and then warmed to 22 °C for 2 hours. Hexane (75 ml) was added, and the white precipitate of 1,3-dicyclohexylurea was removed by filtration. The filtrate was washed with water (50 ml), 1 M hydrochloric acid (50 ml) and 1 M sodium hydroxide (50 mL), and then dried over anhydrous magnesium sulfate. The solvent was removed under vacuum, and the crude citronellyl cyclobutanecarboxylate ester was purified by column chromatography using 9:1 hexane:ethyl acetate as eluent.

^{1}H NMR, and ^{13}C NMR results are shown at the legend of Supplementary Figure 13.

Supplementary References

1. Du, Y. *et al.* Molecular evidence for dual pyrethroid-receptor sites on a mosquito sodium channel. *Proc. Natl. Acad. Sci. U. S. A.* **110**, 11785 LP – 11790 (2013).

2. Neises, B. & Steglich, W. Simple method for the esterification of carboxylic acids. *Angew. Chem. Int. Ed.* **17**, 522-524 (1978).
