# Supplementary material for: A dual-target molecular mechanism of pyrethrum repellency against mosquitoes
Source: Nat Commun. 2021 May 5;12:2553. doi: 10.1038/s41467-021-22847-0 (PMC8099882; doi:10.1038/s41467-021-22847-0)
Supplement: Supplementary file 2 — Reporting Summary [file 41467_2021_22847_MOESM2_ESM.pdf]

nature research

Corresponding author(s): Ke Dong

Last updated by author(s): March 16, 2021

Reporting Summary

Nature Research wishes to improve the reproducibility of the work that we publish. This form provides structure for consistency and transparency in reporting. For further information on Nature Research policies, see our Editorial Policies and the Editorial Policy Checklist.

Statistics

For all statistical analyses, confirm that the following items are present in the figure legend, table legend, main text, or Methods section.

n/a	Confirmed	
☐	☒	The exact sample size (n) for each experimental group/condition, given as a discrete number and unit of measurement
☐	☒	A statement on whether measurements were taken from distinct samples or whether the same sample was measured repeatedly
☐	☒	The statistical test(s) used AND whether they are one- or two-sided<br>*Only common tests should be described solely by name; describe more complex techniques in the Methods section.*
☐	☒	A description of all covariates tested
☐	☒	A description of any assumptions or corrections, such as tests of normality and adjustment for multiple comparisons
☐	☒	A full description of the statistical parameters including central tendency (e.g. means) or other basic estimates (e.g. regression coefficient) AND variation (e.g. standard deviation) or associated estimates of uncertainty (e.g. confidence intervals)
☐	☒	For null hypothesis testing, the test statistic (e.g. F, t, r) with confidence intervals, effect sizes, degrees of freedom and P value noted<br>*Give P values as exact values whenever suitable.*
☒	☐	For Bayesian analysis, information on the choice of priors and Markov chain Monte Carlo settings
☒	☐	For hierarchical and complex designs, identification of the appropriate level for tests and full reporting of outcomes
☒	☐	Estimates of effect sizes (e.g. Cohen's d, Pearson's r), indicating how they were calculated

Our web collection on statistics for biologists contains articles on many of the points above.

Software and code

Policy information about availability of computer code

Data collection	No software were used in data collection
Data analysis	Description of published software used for data analysis is provided and cited in the Methods section (pages 9-14) and figure legends. These include:<br>Prism version 5: https://www.graphpad.com/scientific-software/prism/<br>Autospike version 3.1: http://www.ockenfels-syntech.com/download-2-2/<br>pCLAMP 10 software suite: https://mdc.custhelp.com/app/answers/detail/a_id/18779/~/axon™pclamp™-10-electrophysiology-data-acquisition-%26-analysis-software-download<br>MEGA6: https://www.megasoftware.net/<br>iTOL v5: https://itol.embl.de/<br>Chopchop online tool (http://chopchop.cbu.uib.no/)<br>and Bioedit v7.2.https://bioedit.software.informer.com/7.2/

For manuscripts utilizing custom algorithms or software that are central to the research but not yet described in published literature, software must be made available to editors and reviewers. We strongly encourage code deposition in a community repository (e.g. GitHub). See the Nature Research guidelines for submitting code & software for further information.

Data

Policy information about availability of data

All manuscripts must include a data availability statement. This statement should provide the following information, where applicable:

- Accession codes, unique identifiers, or web links for publicly available datasets
- A list of figures that have associated raw data
- A description of any restrictions on data availability

Vectorbase (www.vectorbase.org); the NCBI website (http://www.ncbi.nlm.nih.gov). The data that support all experimental findings of this study are available within the paper and its Supplementary Information files. Raw data necessary to reproduce all statistical analyses and results in the paper as well as P values for all figures are provided in the source data file. Source data are provided with this paper.

Field-specific reporting

Please select the one below that is the best fit for your research. If you are not sure, read the appropriate sections before making your selection.

- [x] Life sciences
- [ ] Behavioural & social sciences
- [ ] Ecological, evolutionary & environmental sciences

For a reference copy of the document with all sections, see nature.com/documents/nr-reporting-summary-flat.pdf

Life sciences study design

All studies must disclose on these points even when the disclosure is negative.

Sample size	The sample size and the results of statistical analyses are described in the relevant figure legends. Sample size was determined based on experimental trials and with consideration of previous publications on similar experiments [Degennaro, M. et al. Orco mutant mosquitoes lose strong preference for humans and are not repelled by volatile DEET. Nature 498, 487–491 (2013). Boyle, S. M. et al. Natural DEET substitutes that are strong olfactory repellents of mosquitoes and flies. bioRxiv 60178 (2016)] to allow for confident statistical analyses. No statistical methods were used to predetermine sample sizes
Data exclusions	No data that pass quality control were excluded from analysis
Replication	The number of replication for each experiment (at least two repeats, but mostly three times) is described in the relevant figure legends. Results were reproducible in all repeats with the same trend
Randomization	Mosquitoes of different strains were reared side by side to minimize unexpected environmental variations. Female mosquitoes were collected randomly from a large colony for each experiment.
Blinding	Research were not blinded to allocation during experiments and outcome assessment mainly because compounds have distinct smells. We instead have multiple researchers to independently repeat experiments to verify observations.

Reporting for specific materials, systems and methods

We require information from authors about some types of materials, experimental systems and methods used in many studies. Here, indicate whether each material, system or method listed is relevant to your study. If you are not sure if a list item applies to your research, read the appropriate section before selecting a response.

Materials & experimental systems

n/a	Involved in the study	
x		Antibodies
x		Eukaryotic cell lines
x		Palaeontology and archaeology
	x	Animals and other organisms
x		Human research participants
x		Clinical data
x		Dual use research of concern

Methods

n/a	Involved in the study	
x		ChIP-seq
x		Flow cytometry
x		MRI-based neuroimaging

Animals and other organisms

Policy information about studies involving animals; ARRIVE guidelines recommended for reporting animal research

Laboratory animals	Five strains of Aedes aegypti: Orlando, Rockefeller, Orco-/-; ROCK:KDR and AaOr31-/-. One strain of Anopeles gambiae: Kisumu; 4-9 day old female mosquitoes.

Wild animals	The study did not involve wild animals
Field-collected samples	The study did not involve field-collected samples
Ethics oversight	No ethical approval was required. Our University Institutional Biosafety Committee concluded that experimenters were not directly in contact with mosquitoes or chemicals.

Note that full information on the approval of the study protocol must also be provided in the manuscript.
